# The effectiveness of research implementation strategies for promoting evidence-informed policy and management decisions in healthcare: a systematic review

**DOI:** 10.1186/s13012-017-0662-0

**Published:** 2017-11-14

**Authors:** Mitchell N. Sarkies, Kelly-Ann Bowles, Elizabeth H. Skinner, Romi Haas, Haylee Lane, Terry P. Haines

**Affiliations:** 1 Kingston Centre, Monash University and Monash Health Allied Health Research Unit, 400 Warrigal Road, Heatherton, VIC 3202 Australia; 2 Monash University Department of Community Emergency Health and Paramedic Practice, Building H McMahons Road, Frankston, VIC 3199 Australia

**Keywords:** Implementation, Translation, Health, Policy, Management

## Abstract

**Background:**

It is widely acknowledged that health policy and management decisions rarely reflect research evidence. Therefore, it is important to determine how to improve evidence-informed decision-making. The primary aim of this systematic review was to evaluate the effectiveness of research implementation strategies for promoting evidence-informed policy and management decisions in healthcare. The secondary aim of the review was to describe factors perceived to be associated with effective strategies and the inter-relationship between these factors.

**Methods:**

An electronic search was developed to identify studies published between January 01, 2000, and February 02, 2016. This was supplemented by checking the reference list of included articles, systematic reviews, and hand-searching publication lists from prominent authors. Two reviewers independently screened studies for inclusion, assessed methodological quality, and extracted data.

**Results:**

After duplicate removal, the search strategy identified 3830 titles. Following title and abstract screening, 96 full-text articles were reviewed, of which 19 studies (21 articles) met all inclusion criteria. Three studies were included in the narrative synthesis, finding policy briefs including expert opinion might affect intended actions, and intentions persisting to actions for public health policy in developing nations. Workshops, ongoing technical assistance, and distribution of instructional digital materials may improve knowledge and skills around evidence-informed decision-making in US public health departments. Tailored, targeted messages were more effective in increasing public health policies and programs in Canadian public health departments compared to messages and a knowledge broker. Sixteen studies (18 articles) were included in the thematic synthesis, leading to a conceptualisation of inter-relating factors perceived to be associated with effective research implementation strategies. A unidirectional, hierarchal flow was described from (1) establishing an *imperative* for practice change, (2) building *trust* between implementation stakeholders and (3) developing a *shared vision*, to (4) actioning *change mechanisms*. This was underpinned by the (5) employment of effective *communication strategies* and (6) provision of *resources* to support change.

**Conclusions:**

Evidence is developing to support the use of research implementation strategies for promoting evidence-informed policy and management decisions in healthcare. The design of future implementation strategies should be based on the inter-relating factors perceived to be associated with effective strategies.

**Trial registration:**

This systematic review was registered with Prospero (record number: 42016032947).

**Electronic supplementary material:**

The online version of this article (10.1186/s13012-017-0662-0) contains supplementary material, which is available to authorized users.

## Background

The use of research evidence to inform health policy is strongly promoted [1]. This drive has developed with increased pressure on healthcare organisations to deliver the most effective health services in an efficient and equitable manner [2]. Policy and management decisions influence the ability of health services to improve societal outcomes by allocating resources to meet health needs [3]. These decisions are more likely to improve outcomes in a cost-efficient manner when they are based on the best available evidence [4–8].

Evidence-informed decision-making refers to the complex process of considering the best available evidence from a broad range of information when delivering health services [1, 9, 10]. Policy and management decisions can be influenced by economic constraints, community views, organisational priorities, political climate, and ideological factors [11–16]. While these elements are all important in the decision-making process, without the support of research evidence they are an insufficient basis for decisions that affect the lives of others [17, 18].

Recently, increased attention has been given to implementation research to reduce the gap between research evidence and healthcare decision-making [19]. This growing but poorly understood field of science aims to improve the uptake of research evidence in healthcare decision-making [20]. Research implementation strategies such as knowledge brokerage and education workshops promote the uptake of research findings into health services. These strategies have the potential to create systematic, structural improvements in healthcare delivery [21]. However, many barriers exist to successful implementation [22, 23]. Individuals and health services face financial disincentives, lack of time or awareness of large evidence resources, limited critical appraisal skills, and difficulties applying evidence in context [24–30].

It is important to evaluate the effectiveness of implementation strategies and the inter-relating factors perceived to be associated with effective strategies. Previous reviews on health policy and management decisions have focussed on implementing evidence from single sources such as systematic reviews [29, 31]. Strategies that involved simple written information on accomplishable change may be successful in health areas where there is already awareness of evidence supporting practice change [29]. Re-conceptualisation or improved methodological rigor has been suggested by Mitton et al. to produce a richer evidence base for future evaluation, however only one high-quality randomised controlled trial has been identified since [9, 32, 33]. As such, an updated review of emerging research in this topic is needed to inform the selection of research implementation strategies in health policy and management decisions.

The primary aim of this systematic review was to evaluate the effectiveness of research implementation strategies for promoting evidence-informed policy and management decisions in healthcare. A secondary aim of the review was to describe factors perceived to be associated with effective strategies and the inter-relationship between these factors.

## Methods

### Identification and selection of studies

This systematic review was registered with Prospero (record number: 42016032947) and has been reported consistent with the Preferred Reporting Items for Systematic Reviews and Meta-Analysis (PRISMA) guidelines (Additional file 1). Ovid MEDLINE, Ovid EMBASE, PubMed, CINAHL Plus, Scopus, Web of Science Core Collection, and The Cochrane Library were searched electronically from January 01, 2000, to February 02, 2016, in order to retrieve literature relevant to the current healthcare environment. The search was limited to the English language, and terms relevant to the field, population, and intervention were combined (Additional file 2). Search terms were selected based on their sensitivity, specificity, validity, and ability to discriminate implementation research articles from non-implementation research articles [34–36]. Electronic database searches were supplemented by cross-checking the reference list of included articles and systematic reviews identified during the title and abstract screening. Searches were also supplemented by hand-searching publication lists from prominent authors in the field of implementation science.

### Study selection

#### Type of studies

All study designs were included. Experimental and quasi-experimental study designs were included to address the primary aim. No study design limitations were applied to address the secondary aim.

#### Population

The population included individuals or bodies who made resource allocation decisions at the managerial, executive, or policy level of healthcare organisations or government institutions. Broadly defined as healthcare policy-makers or managers, this population focuses on decision-making to improve population health outcomes by strengthening health systems, rather than individual therapeutic delivery. Studies investigating clinicians making decisions about individual clients were excluded, unless these studies also included healthcare policy-makers or managers.

#### Interventions

Interventions included research implementation strategies aimed at facilitating evidence-informed decision-making by healthcare policy-makers and managers. Implementation strategies may be defined as methods to incorporate the systematic uptake of proven evidence into decision-making processes to strengthen health systems [37]. While these interventions have been described differently in various contexts, for the purpose of this review, we will refer to these interventions as ‘research implementation strategies’.

#### Type of outcomes

This review focused on a variety of possible outcomes that measure the use of research evidence. Outcomes were broadly categorised based on the four levels of Kirkpatrick’s Evaluation Model Hierarchy: level 1—reaction (e.g. change in attitude towards evidence), level 2—learning (e.g. improved skills acquiring evidence), level 3—behaviour (e.g. self-reported action taking), and level 4—results (e.g. change in patient or organisational outcomes) [38].

### Screening

The web-based application Covidence (Covidence, Melbourne, Victoria, Australia) was used to manage references during the review [39]. Titles and abstracts were imported into Covidence and independently screened by the lead investigator (MS) and one of two other reviewers (RH, HL). Duplicates were removed throughout the review process using Endnote (EndNote^™^, Philadelphia, PA, USA), Covidence and manually during reference screening. Studies determined to be potentially relevant or whose eligibility was uncertain were retrieved and imported to Covidence for full-text review. The lead investigator (MS) and one of two other reviewers (RH, HL) then independently assessed the full-text articles for the remaining studies to ascertain eligibility for inclusion. A fourth reviewer (KAB) independently decided on inclusion or exclusion if there was any disagreement in the screening process. Attempts were made to contact authors of studies whose full-text articles were unable to be retrieved, and those that remained unavailable were excluded.

### Quality assessment

Experimental study designs, including randomised controlled trials and quasi-experimental studies, were independently assessed for risk of bias by the lead investigator (MS) and one of two other reviewers (RH, HL) using the Cochrane Collaboration’s tool for assessing risk of bias [40]. Non-experimental study designs were independently assessed for risk of bias by the lead investigator (MS) and one of two other reviewers (RH, HL) using design-specific risk-of-bias-critical appraisal tools: (1) Quality Assessment Tool for Observational Cohort and Cross-Sectional Studies from the National Heart, Lung, and Blood Institute (NHLBI; [41], February) and (2) Critical Appraisal Skills Program (CASP) Qualitative Checklist for qualitative, case study, and evaluation designs [42].

### Data extraction

Data was extracted using a standardised, piloted data extraction form developed by reviewers for the purpose of this study (Additional file 3). The lead investigator (MS) and one of two other reviewers (RH, HL) independently extracted data relating to the study details, design, setting, population, demographics, intervention, and outcomes for all included studies. Quantitative results were also extracted in the same manner from experimental studies that reported quantitative data relating to the effectiveness of research implementation strategies in promoting evidence-informed policy and management decisions in healthcare. Attempts were made to contact authors of studies where data was not reported or clarification was required. Disagreement between investigators was resolved by discussion, and where agreement could not be reached, an independent fourth reviewer (KAB) was consulted.

### Data analysis

A formal meta-analysis was not undertaken due to the small number of studies identified and high levels of heterogeneity in study approaches. Instead, a narrative synthesis of experimental studies evaluating the effectiveness of research implementation strategies for promoting evidence-informed policy and management decisions in healthcare and a thematic synthesis of non-experimental studies were performed to describe factors perceived to be associated with effective strategies and the inter-relationship between these factors. Experimental studies were synthesised narratively, defined as studies reporting quantitative results with both an experimental and comparison group. This included specified quasi-experimental designs, which report quantitative before and after results for primary outcomes related to the effectiveness of research implementation strategies for promoting evidence-informed policy and management decisions in healthcare. Non-experimental studies were synthesised thematically, defined as studies reporting quantitative results without both an experimental and control group, or studies reporting qualitative results. This included quasi-experimental studies that do not report quantitative before and after results for primary outcomes related to the effectiveness of research implementation strategies for promoting evidence-informed policy and management decisions in healthcare.

The thematic synthesis was informed by inductive thematic approach for data referring to the factors perceived to be associated with effective strategies and the inter-relationship between these factors. The thematic synthesis in this systematic review was based on methods described by Thomas and Harden [43]. Methods involved three stages of analysis: (1) line-by-line coding of text, (2) inductive development of descriptive themes similar to those reported in primary studies, (3) analytical themes representing new interpretive constructs undeveloped within studies but apparent between studies once data is synthesised. Data reported in the results section of included studies were reviewed line-by-line and open coded according to meaning and content by the lead investigator (MS). Codes were developed using an inductive approach by the lead investigator (MS) and a second reviewer (TH). Concurrent with data analysis, this entailed constant comparison, ongoing development, and comparison of new codes as each study was coded. Immersing reviewers in the data, reflexive analysis, and peer debriefing techniques were used to ensure methodological rigor throughout the process. Codes and code structure was considered finalised at point of theoretical saturation (when no new concepts emerged from a study). A single researcher (MS) was chosen to conduct the coding in order to embed the interpretation of text within a single immersed individual to act as an instrument of data curation [44, 45]. Simultaneous axial coding was performed by the lead investigator (MS) and a second reviewer (TH) during the original open coding of data to identify relationships between codes and organise coded data into descriptive themes. Once descriptive themes were developed, the two investigators then organised data across studies into analytical themes using a deductive approach by outlining relationships and interactions between codes across studies. To ensure methodological rigor, a third reviewer (JW) was consulted via group discussion to develop final consensus. The lead author (MS) reviewed any disagreements in descriptive and analytical themes by returning to the original open codes. This cyclical process was repeated until themes were considered to sufficiently describe the factors perceived to be associated with effective strategies and the inter-relationship between these factors.

## Results

### Search results

**Fig. 1:**
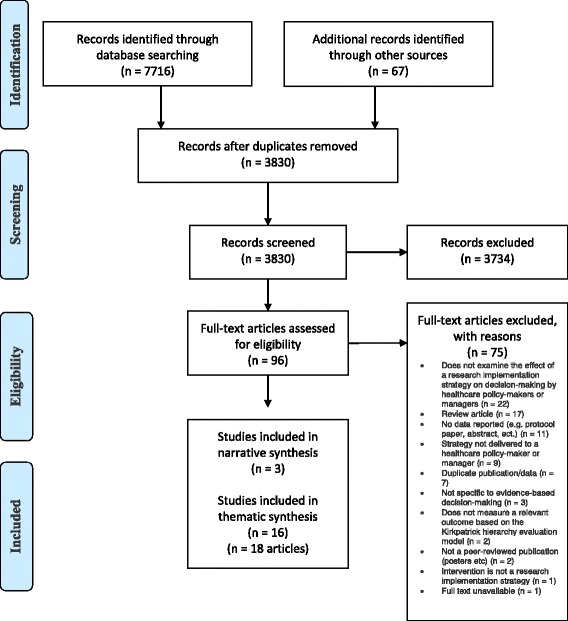
PRISMA Flow Diagram

The search strategy identified a total of 7783 articles, 7716 were identified by the electronic search strategy, 56 from reference checking of identified systematic reviews, 8 from reference checking of included articles, and 3 articles from hand-searching publication lists of prominent authors. Duplicates (3953) were removed using Endnote (*n* = 3906) and Covidence (*n* = 47), leaving 3830 articles for screening (Fig. 1).

Of the 3830 articles, 96 were determined to be potentially eligible for inclusion after title and abstract screening (see Additional file 4 for the full list of 96 articles). The full-text of these 96 articles was then reviewed, with 19 studies (*n* = 21 articles) meeting all relevant criteria for inclusion in this review [9, 27, 46–64]. The most common reason for exclusion upon full-text review was that articles did not examine the effect of a research implementation strategy on decision-making by healthcare policy-makers or managers (*n* = 22).

### Characteristics of included studies

The characteristics of included studies are shown in Table 1. Three experimental studies evaluated the effectiveness of research implementation strategies for promoting evidence-informed policy and management decisions in healthcare systems. Sixteen non-experimental studies described factors perceived to be associated with effective research implementation strategies.

**Table 1:** Characteristics of included studies

Author, year, country	Study design	Health topic	Health organisation setting	Decision-maker population	Control group	Research implementation group			Outcome measure
Beynon et al. 2012, multi-national [46]	Randomised controlled trial	Health in low- and middle-income countries	Public health	Professions from government and non-government organisations and academia (*n* = 807)	Existing Institute of Development Studies publication from the In Focus Policy Briefing series	Basic 3-page policy brief	Basic 3-page policy brief plus an expert opinion piece	Basic 3-page policy brief plus an unnamed research fellow opinion piece	Online questionnaires (immediately, 1 week and 3 months post) Semi-structured interviews (in-between 1 week and 3 months and after 3 month questionnaires)
Brownson et al. 2007, USA [47]	Quasi-experimental	Guidelines for promoting physical activity	State and local health departments (*n* = 8)	Health department program managers, administrators, division, bureau, or agency heads, and ‘other’ positions e.g. program planner, nutritionist (State *n* = 58) (Local *n* = 55) (Other *n* = 80)	Remaining states and the Virgin Islands served as the comparison group)	Workshops, ongoing technical assistance and distribution of an instructional CD-ROM			25-item questionnaire survey (2 years)
Bullock et al. 2012, UK [48]	Programme evaluation case study	Non-specific	NHS health service delivery organisations (*n* = 10)	Management fellows (*n* = 11) Chief investigators (*n* = 10) Additional co-applicants from the research teams (*n* = 3) Workplace line-managers (*n* = 12) (Total *n* = 36)	None	UK Service Delivery and Organisation (SDO) Management Fellowship programme			Semi-structured face-to-face interviews
Campbell et al. 2011, Australia [49]	Program evaluation	Range of topics related to population health, health services organisation and delivery, and cost effectiveness	State-level policy agencies, including both the New South Wales and Victorian Departments of Health (*n* = 5)	Policymakers (*n* = 8)	None	‘Evidence check’ rapid policy relevant review and knowledge brokers			Structured interviews (2–3 years)
Chambers et al. 2012, UK [58]	Case study	Adolescents with eating disorders	Primary care	Local NHS commissioners and clinicians (*n* = 15)	None	Contextualised evidence briefing based on systematic review			Short evaluation questionnaire
Champagne et al. 2014, Canada [59]	Case studies	Non-specific	Academic health centres (*n* = 6)	Extra fellows, SEARCHers, Colleagues, Supervisors, Vice-presidents and CEOs (*n* = 84)	None	Executive Training for Research Application (EXTRA) program Swift, Efficient, Application of Research in Community Health (SEARCH) Classic program			Semi-structured interviews and data from available organisational documents
Courtney et al. 2007, USA [60]	Cohort study	Substance abuse treatment programs	Community-based treatment units (*n* = 53 units from *n* = 24 multisite parent organisations)	Directors and clinical supervisors (*n* = 309)	None	2-day workshop (entitled “TCU Model Training-making it real”)			Compliance with early steps of consulting and planning activities (1 month) Organisational Readiness for Change (ORC) assessment (1 month)
Dagenais et al. 2015, Burkina Faso [52]	Implementation evaluation	Maternal health, malaria prevention, free healthcare, and family planning	Public health	Researchers; Knowledge brokers; health professionals; community-based organisations; and local, regional, and national policy-makers (*n* = 47)	None	Knowledge broker			Semi-structured individual interviews and participant training session questionnaires
Dobbins et al. 2001, Canada [61].	Cross-sectional follow-up survey	Home visiting as a public health intervention, community-based heart health promotion, adolescent suicide prevention, community development, and parent-child health	Public health units (*n* = 41)	Public health decision-makers (*n* = 147)	None	Systematic reviews			Cross-sectional follow-up telephone survey
Dobbins et al. 2009, Canada [9]	Randomised controlled trial	Promotion of healthy bodyweight in children	Public health departments (*n* = 108)	Front-line staff 35% Managers 26% Directors 10% Coordinators 9% Other 20% (*n* = 108)	Access to an online registry of research evidence	Tailored, targeted messages Access to an online registry of research evidence	Knowledge broker Tailored, targeted messages Access to an online registry of research evidence		Telephone-administered survey (knowledge transfer and exchange data collection tool) 1–3 months post completion of intervention (intervention lasted 12 months)
Dopp et al. 2013, Netherlands [55]	Mixed methods process evaluation	Dementia	Home-based community health	Managers (*n* = 20) Physicians (*n* = 36) Occupational therapists (*n* = 36)	None	Multifaceted implementation strategy			Semi-structured telephone interviews with managers (3–5 months) Semi-structured focus groups with occupational therapists (2 months)
Flanders et al. 2009, USA [53]	Implementation evaluation	Patient safety	Teaching and nonteaching, urban and rural, government and private, as well as academic and community settings (*n* = 9)	Hospitalists or quality improvement staff, representatives from each institutions department of quality or department of patient safety (*n* = 9)	None	The Hospitalists as Emerging Leaders in Patient Safety (HELPS) Consortium			Web-based survey (post meetings)
Gagliardi et al. 2008, Canada [56]	Mixed methods exploratory	Colorectal cancer	Not specified	Researchers (*n* = 6) Clinicians (*n* = 13) Manager (*n* = 5) Policy-maker (*n* = 5) (Total *n* = 29)		Review of Canadian health services research in colorectal cancer based on published performance measures 1-day workshop to prioritise research gaps, define research questions and plan implementation of a research study.			Participant survey (prior to workshop) Observation of workshop participants (during workshop) Semi-structured interviews and observation of workshop participants (during workshop)
Kitson et al. 2011, Australia [50]	Project evaluation	7 clinical topic areas identified in The Older Person and Improving Care (TOPIC7) project	Large tertiary hospital (*n* = 1)	Clinical nursing leaders (*n* = 14) Team members (*n* = 28) Managers (*n* = 11)	None	Knowledge translation toolkit			Semi-structured interviews and questionnaires
Moat et al. 2014, multi-national, [63]	Survey evaluation	Health in low- and middle-income countries	Public health	Policy-makers, stakeholders and researchers (*n* = 530)	None	Evidence briefs Deliberative dialogues			Questionnaire surveys
Traynor et al. 2014, Canada [57]	Single mixed-methods study and a case study	Child obesity	Canadian public health departments (*n* = 30) (Case studies *n* = 3)	Health department staff (RCT *n* = 108) (Case A *n* = 258) (Case B *n* = 391) (Case C *n* = 155)	Access to an online registry of research evidence	Knowledge brokering			Knowledge broker journaling (baseline, interim, follow-up) Qualitative interviews *n* = 12 (1 year) Case study interviews *n* = 37 (baseline, interim and 22 month follow-up)
Uneke et al. 2015, Nigeria [54]	Implementation evaluation	Low- and middle-income country health	Public health	Directors from Ministry of Health (*n* = 9) Senior researchers from the university (*n* = 5) NGO executive director (*n* = 1) Director of public health in the local government service commission (*n* = 1) Executive secretary of the AIDS control agency (*n* = 1) State focal person of Millennium Development Goals (*n* = 1) (Total *n* = 18)	None	Training workshop (HPAC) Certificate course (HPAC) Policy brief and hosting of a multi-stakeholder policy dialogue (HPAC)			Semi-structured interviews (end of each intervention) Group discussions
Waqa et al. 2013, Fiji [51]	Process evaluation	Overweight and obesity	Public health government organisations (*n* = 6) NGOs (*n* = 2)	Senior managers (*n* = 20) Middle managers (*n* = 22) Junior managers (*n* = 7) (Total *n* = 49)	None	Policy brief and hosting of a multi-stakeholder policy dialogue (HPAC)			Semi-structured interviews Process diaries
Wilson et al. 2015, Canada [64]	Process evaluation	Non specific	Policy analysts (*n* = 9) Health department units (*n* = 6)	Senior analysts (*n* = 8) Junior analysts (*n* = 1)	None	Access to an online registry of research evidence			Semi-structured telephone interviews

#### Study design

Of the 19 included studies, there were two randomised controlled trials (RCTs) [9, 46], one quasi-experimental study [47], four program evaluations [48–51], three implementation evaluations [52–54], three mixed methods [55–57], two case studies [58, 59], one survey evaluation [63], one process evaluation [64], one cohort study [60], and one cross-sectional follow-up survey [61].

#### Participants and settings

The largest number of studies were performed in Canada (*n* = 6), followed by the United States of America (USA) (*n* = 3), the United Kingdom (UK) (*n* = 2), Australia (*n* = 2), multi-national (*n* = 2), Burkina Faso (*n* = 1), the Netherlands (*n* = 1), Nigeria (*n* = 1), and Fiji (*n* = 1). Health topics where research implementation took place were varied in context. Decision-makers were typically policy-makers, commissioners, chief executive officers (CEOs), program managers, coordinators, directors, administrators, policy analysts, department heads, researchers, change agents, fellows, vice presidents, stakeholders, clinical supervisors, and clinical leaders, from the government, academia, and non-government organisations (NGOs), of varying education and experience.

#### Research implementation strategies

There was considerable variation in the research implementation strategies evaluated, see Table 2 for summary description. These strategies included knowledge brokering [9, 49, 51, 52, 57], targeted messaging [9, 64], database access [9, 64], policy briefs [46, 54, 63], workshops [47, 54, 56, 60], digital materials [47], fellowship programs [48, 50, 59], literature reviews/rapid reviews [49, 56, 58, 61], consortium [53], certificate course [54], multi-stakeholder policy dialogue [54], and multifaceted strategies [55].

**Table 2:** Implementation strategy summary description

Study (author, year)	Implementation strategy	Theoretical framework	Summary description
Dobbins 2009, [57, 9]	Access to online registry of research evidence	Dobbins framework	Reference offered a link to a short summary and full text of each review
	Tailored, targeted messages and access to online registry of research evidence		Title of systematic review and link to full reference, including abstract sent via email Reference offered a link to a short summary and full text of each review
	Knowledge broker, tailored messages, and access to online registry of research evidence		Knowledge brokers ensured relevant evidence was transferred in useful ways to decision-makers to assist skills and capacity development for translating evidence into local healthcare delivery. Activities included regular electronic and telephone communication, one face-to-face site visit, and invitation to a workshop. Title of systematic review and link to full reference, including abstract sent via email Reference offered a link to a short summary and full text of each review
Beynon 2012, [46]	Basic 3-page policy brief	A simple theory of change for a policy brief	Link to policy brief sent via email
	Basic 3-page policy brief plus an expert opinion piece		Same basic 3-page policy brief plus an expert opinion piece credited and written by a sector expert, Lawrence Haddad. Link to policy brief sent via email
	Basic 3-page policy brief plus an un-credited expert opinion piece		Same basic 3-page policy brief and expert opinion piece but credited to an unnamed research fellow. Link to policy brief sent via email
Brownson 2007, [47]	Workshops, ongoing technical assistance, and distribution of an instructional digital materials	Framework for a systematic approach to promoting effective physical activity programs and policies	Workshops included: formal presentations, case study applications, and ‘real-world’ examples Ongoing technical assistance included: strategic planning, grant writing, tuition waivers, consultation for effective strategy planning, and dissemination guidance Digital materials included: additional information, prominent public health leader interviews, and resource tools
Courtney 2007, [60]	Workshop	The change book	Pre-workshop completion of organisational readiness for change assessment. Workshop included: conceptual overview presentations, personalised feedback, comparison with other agencies, and group work
Bullock 2012 [48]	Fellowship program	Programme evaluation framework (adapted from Kirkpatrick)	Practicing managers work within research teams for the duration of a funded project
Campbell 2011, [49]	‘Evidence check’ rapid policy relevant review and knowledge brokers	Van Kammen et al.’s approach to knowledge brokering	Pre-meeting commissioning tool completed prior to knowledge broker meetings, which clarified research question. Then a rapid review summary of evidence on policy area is performed
Chambers 2012, [58]	Contextualised evidence briefing based on systematic review	Facilitators of the use of research evidence identified by a systematic review (Innvaer et al. [28])	Researcher attended meeting to clarify research question and prepared a concise evidence briefing on policy area
Champagne 2014, [59]	Executive Training for Research Application (EXTRA) program	Knowledge creation logic model	Program included: residency sessions, projects, educational activities, networking, and post-program activities
	Swift, Efficient, Application of Research in Community Health (SEARCH) Classic program		Program included: modules, inter-module work, and application of knowledge to practice-based projects
Dagenais 2015, [52]	Knowledge broker	Theoretical models for understanding health behaviour	Knowledge broker tasks included: liaison, information management and support, partner meetings, developing documentary research strategies, database set-up for relevant information, drafting summary documents, workshops, and developing and monitoring actions plans
Dobbins 2001, [61]	Systematic reviews	–	Systematic reviews of the effectiveness of public health interventions disseminated to public health decision-makers
Dopp 2013, [55]	Multifaceted implementation strategy	The model of Grol and Wensing	Educational materials, educational meetings, outreach visits, newsletters, and reminders
Flanders 2009, [53]	The Hospitalists as Emerging Leaders in Patient Safety (HELPS) Consortium	–	Meetings on quality improvement methodology and substantiative patient safety-related topics, and a final half-day session drawing out learning’s and next steps
Gagliardi 2008, [56]	Comprehensive review and workshop	Author’s conceptual model of factors influencing effectiveness of knowledge exchange	Comprehensive review of Canadian health services research in colorectal cancer based on published performance measures and workshop to prioritise research gaps, define research questions, and plan implementation of a research study
Kitson 2011, [50]	Knowledge translation toolkit	–	Team recruitment, clarification, stakeholder engagement, pre-strategy evaluation, training, support meetings, communication and feedback, process evaluation, dissemination (e.g. posters and presentations), future planning, and program evaluation
Moat et al. 2014, multi-national, [50]	Evidence briefs	Theory of planned behaviour	Evidence briefs and deliberative dialogues across a range of issues and low- and middle-income countries
	Deliberative dialogues		
Uneke 2015, [54]	Training, workshop, certificate course, policy brief, and hosting of a multi-stakeholder policy dialogue	–	Workshop featuring training on the role of research evidence, preparation of policy briefs, how to organise and use policy dialogues, and how to set priorities. Certificate course aimed to foster research capacity, leadership, enhance capacity for evidence-informed decision-making, and health policy monitoring/evaluation. Policy briefs were produced, and the multi-stakeholder policy dialogue between key stakeholders was then held
Waqa 2013, [51, 62]	Knowledge broker capacity building	–	Knowledge coordinated organisation recruitment, mapping policy environment, analysed organisational capacity and support for evidence-informed policymaking, developed evidence-informed policymaking skills, and facilitated development of evidence-informed policy briefs
Wilson et al. 2015, Canada [64]	Access to online registry of research evidence	Framework for assessing country-level efforts to link research to action	The ‘self-serve’ evidence service consisted only of database access
	Access to online registry of research evidence, email alerts, and full-text availability		The ‘full-serve’ evidence service included (1) database access for research evidence addressing questions about governance, financial and delivery arrangements within which programs, services and drugs are provided and about implementation strategies; (2) monthly email alerts about new additions to the database; and (3) full-text article availability

### Quality/risk of bias

#### Experimental studies

The potential risk of bias for included experimental studies according to the Cochrane Collaboration tool for assessing risk of bias is presented in Table 3. None of the included experimental studies reported methods for allocation concealment, blinding of participants and personnel, and blinding of outcome assessment [9, 46, 47]. Other potential sources of bias were identified in each of the included experimental studies including (1) inadequate reporting of *p* values for mixed-effects models, results for hypothesis two, and comparison of health policies and programs (HPP) post-intervention on one study [9], (2) pooling of data from both intervention and control groups limited ability to evaluate the success of the intervention in one study [47], and (3) inadequate reporting of analysis and results in another study [46]. Adequate random sequence generation was reported in two studies [9, 46] but not in one [47]. One study reported complete outcome data [9]; however, large loss to follow-up was identified in two studies [46, 47]. It was unclear whether risk of selective reporting bias was present for one study [46], as outcomes were not adequately pre-specified in the study. Risk of selective reporting bias was identified for one study that did not report *p* values for sub-group analysis [9] and another that only reported change scores for outcome measures [47].

**Table 3:**
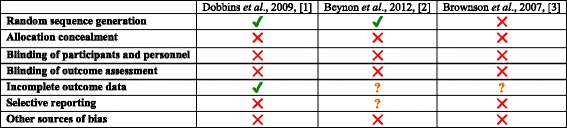
Risk of bias of included experimental studies using the Cochrane Collaboration tool for assessing risk of bias

#### Non-experimental studies

The potential risk of bias for included non-experimental studies according to the Quality Assessment Tool for Observational Cohort and Cross-Sectional Studies from the National Heart, Lung, and Blood Institute, and the Critical Appraisal Skills Program (CASP) Qualitative Checklist is presented in Tables 4 and 5.

**Table 4:**
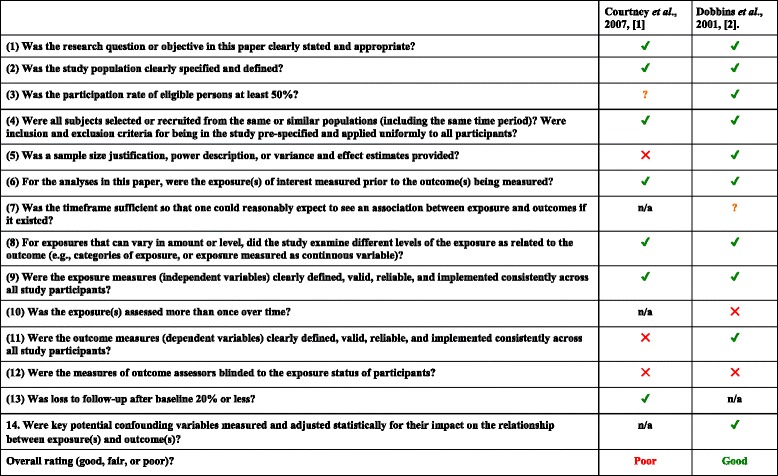
Risk of bias of included non-experimental studies using the Quality Assessment Tool for Observational Cohort and Cross-Sectional Studies

*n/a* not applicable

**Table 5:**
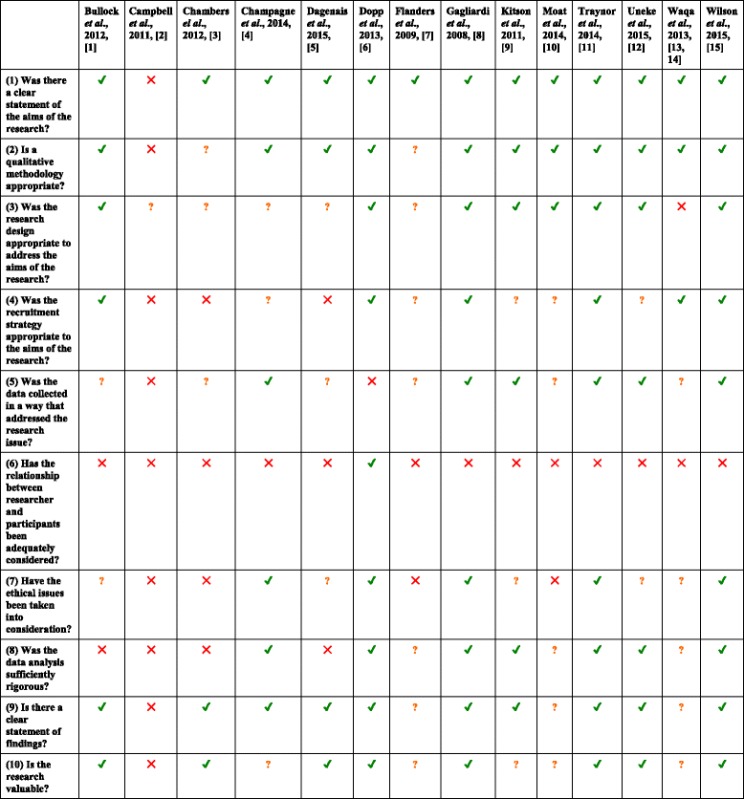
Risk of bias of included non-experimental studies using the Critical Appraisal Skills Program (CASP) Qualitative Checklist

### Narrative synthesis results: effectiveness of research implementation strategies for promoting evidence-informed policy and management decisions in healthcare

Definitive estimates of implementation strategy effect are limited due to the small number of identified studies, and heterogeneity in implementation strategies and reported outcomes. A narrative synthesis of results is described for changes in reaction/attitudes/beliefs, learning, behaviour, and results. See Table 6 for a summary of study results.

**Table 6:** Summary of study results

Study (author, year)	Implementation strategy	Level 1: change in reaction/attitudes/beliefs	Level 2: learning	Level 3: behaviour
Randomised controlled trial				
Beynon 2012 [46]	Basic 3-page policy brief	High-quality ratings Opinion about evidence strength or intervention effectiveness varies by health topic	–	Less likely to source other information and research related to the topic than control
	Basic 3-page policy brief plus an expert opinion piece	High-quality rating Opinion about evidence strength or intervention effectiveness varies by health topic. Increased intention to send policy brief to someone else and tell someone about key messages	–	Less likely to source other information and research related to the topic than control. Trend towards intentions persisting to actions. More likely to send policy brief to someone else
	Basic 3-page policy brief plus an un-credited expert opinion piece	High-quality rating Opinion about evidence strength or intervention effectiveness varies by health topic	–	Less likely to source other information and research related to the topic than control
Dobbins 2009 [9]	Tailored, targeted messages	–	–	Improved use of public health policies and programs compared to control
	Tailored, targeted messages plus a knowledge broker	–	–	Addition of knowledge broker potentially reduced use of public health policies and programs. However, improvements may have occurred in organisations with low research culture
Non-randomised controlled trial				
Brownson 2007 [47]	Workshops, ongoing technical assistance, and digital resources	Change in whether heard of recommendations and attended training. Less likely to report state legislators were supportive of physical activity interventions. No change in other outcomes from baseline	All knowledge and skill measurements improved. Change larger for local than state health department decision-makers in every category except methods in understanding cost. The largest change related to attitudes	Improvement in self-reported individual adapted health behaviour change. No difference in other behaviour change outcomes

#### Randomised controlled trials

Interestingly, the policy brief accompanied by an expert opinion piece was thought to improve both level 1 change in reaction/attitudes/beliefs and level 3 behaviour change outcomes. This was referred to as an “authority effect” [46]. Tailored targeted messages also reportedly improved level 3 behaviour change outcomes. However, the addition of a knowledge broker to this strategy may have been detrimental to these outcomes. When organisational research culture was considered, health departments with low research culture may have benefited from the addition of a knowledge broker, although no *p* values were provided for this finding [9].

#### Non-randomised studies

The effect of workshops, ongoing technical assistance, and distribution of instructional digital materials on level 1 change in reaction/attitudes/beliefs outcomes was difficult to determine, as many measures did not change from baseline scores and the direction of change scores was not reported. However, a reduction in perceived support from state legislators for physical activity interventions was reported after the research implementation strategy. All level 2 learning outcomes were reportedly improved, with change scores larger for local than state health department decision-makers in every category except methods in understanding cost. Results were then less clear for level 3 behaviour change outcomes. Only self-reported individual-adapted health behaviour change was thought to have improved [47].

### Thematic synthesis results: conceptualisation of factors perceived to be associated with effective strategies and the inter-relationship between these factors

**Fig. 2:**
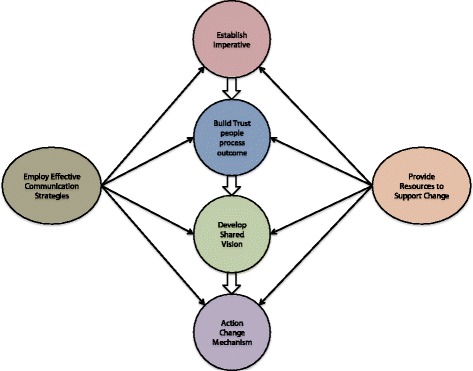
Conceptualisation of Inter-related themes (analytic themes) associated with effective strategies and the inter-relationship between these factors

Due to the relative paucity of evidence for effectiveness studies, a thematic synthesis of non-experimental studies was used to explore the factors perceived to be associated with effective strategies and the inter-relationship between these factors. Six broad, interrelated, analytic themes emerged from the thematic synthesis of data captured in this review (Fig. 2). We developed a conceptualisation of how these themes interrelated from data captured both within and across studies. Some of these analytic themes were specifically mentioned in individual papers, but none of the papers included in this review identified all, nor developed a conceptualisation of how they interrelated. The six analytic themes were conceptualised as having a unidirectional, hierarchal flow from (1) establishing an *imperative* for practice change, (2) building *trust* between implementation stakeholders, (3) developing a *shared vision*, and (4) actioning *change mechanisms*. These were underpinned by (5) employment of effective *communication strategies* and (6) provision of *resources* to support change.

#### Establish imperative

Organisations and individuals were driven to implement research into practice when there was an imperative for practice change. Decision-makers wanted to know why change was important to them, and their organisation and or community. Imperatives were seen as drivers of motivation for change to take place and were evident both internal to the decision-maker (personal gain) and external to the decision-makers (organisational and societal gain).

##### Personal gain

Individuals were motivated to participate in research implementation projects where they could derive personal gain [48, 50, 56]. Involvement in research was viewed as an opportunity rather than an obligation [56]. This was particularly evident in one study by Kitson et al. where all nursing leaders unanimously agreed the potential benefit of supported, experiential learning was substantial, with 13 of 14 committing to leading further interdisciplinary, cross-functional projects [50].

##### Organisational and societal gain

Decision-makers supported research implementation efforts when they aligned to an organisational agenda or an area where societal health needs were identified [48, 50, 53, 55, 59, 64]. Practice change was supported if it was deemed important by decision-makers and aligned with organisational priorities, where knowledge exchange was impeded if changes had questionable relevance to the workplace [48, 53, 64]. Individuals reported motivation to commit to projects they felt would address community needs. For example, in one study, nursing leaders identified their passion for health topics as a reason to volunteer in a practice change process [50]. In another study, managers were supportive of practice change to improve care of people with dementia, as they thought this would benefit the population [55].

#### Build trust

Relationships, leadership authority, and governance constituted the development of trust between stakeholder groups.

##### Relationships

The importance of trusting relationships between managers, researchers, change agents, and staff was emphasised in a number of studies [48, 50, 54, 59, 64]. Developing new relationships through collaborative networking and constant contact reportedly addressed mutual mistrust between policy-makers and the researchers, and engaged others to change practice [54, 59]. Bullock et al. described how pre-existing personal and professional relationships might facilitate implementation strategy success through utilising organisational knowledge and identifying workplace “gatekeepers” to engagement with. In the same study, no real link between healthcare managers and academic resources was derived from fellows that were only weakly connected to healthcare organisations [48].

##### Leadership authority

The leadership authority of those involved in research implementation influenced the development of trust between key stakeholders [50, 52, 55, 59, 61]. Dagenais et al. found recommendations and information was valued if credited from researchers and change agents whose input was trusted [52]. The perception that individuals with senior organisational roles reduce perceived risk and resistance to change was supported by Dobbins et al., who reported that seniority of individuals is a predictor of systematic review use in decision-making [50, 59, 61]. However, professional seniority should be related to the research implementation context, as the perceived lack of knowledge in content area was a barrier to providing managerial support [55].

##### Governance

A number of studies expressed the importance of consistent and sustained executive support in order to maintain project momentum [48, 50, 52, 53, 59, 64]. In the study by Kitson et al.*,* individuals expressed concern and anxiety around reputational risk if consistent organisation support was not provided [50]. Organisational capacity was enhanced with strong management support and policies [57]. Uneke et al. identified good stewardship in the form of governance to provide accountability and protection for individuals and organisations in their study. Participants in this study unanimously identified the need for performance measurement mechanisms for the health policy advisory committee to promote sustainability and independent evidence to policy advice [54]. Bullock et al. found that managers view knowledge exchange in a transaction manner and are keen to know and use project results as soon as possible. However, researchers and change agents may not wish to apply results due to the phase of the project [48]. This highlighted the importance of governance systems to support confidentiality and limiting the release of project results before stakeholders are confident of findings.

#### Develop shared vision

A shared vision for desired change and outcomes can be built around common goal through improving understanding, influencing behaviour change, and working with the characteristics of organisations.

##### Stakeholder understanding

Improving the understanding of research implementation was considered a precursor to building shared vision [50, 52, 55, 56]. Policy-makers reported lack of time prevented them from performing an evidence review and desired experientially tailored information, education, and avoidance of technical language to improve understanding [52, 55, 58]. It was perceived that lack of clarity limited project outcomes in the study by Gagliardi et al., which emphasised the need for simple processes [56]. When challenges arose in Kitson et al.*,* ensuring all participants understood their role from implementation outset was suggested as a process improvement [50].

##### Influence change

Knowledge brokers in Campbell et al. were able to elicit well-defined research questions if they were open, honest, and frank in their approach to policy-makers. Policy-makers felt that knowledge brokering was more useful for shaping parameters, scope, budget, and format of projects, which provides guidance for decision-making rather than being prescriptive [49]. However, conclusive recommendations that aim for a consensus are viewed favourably by policy-makers, which means a balance between providing guidance without being too prescriptive, must be achieved [63]. Interactive strategies may allow change agents to gain better understanding of evidence in organisational decisions and guide attitudes towards evidence-informed decision-making. Champagne et al. observed fellows participating in this interactive, social process, and Dagenais et al. reported practical exercises and interactive discussions were appreciated by knowledge brokers in their own training [52, 59]. Another study reported barriers in work practice challenges being viewed as criticism; despite this, organisation staff valued leaders’ ability to inspire a shared vision and identified ‘challenging processes’ as the most important leadership practice [50].

##### Characteristics of organisation

Context-specific organisational characteristics such as team dynamics, change culture, and individual personalities can influence the effectiveness of research implementation strategies [50, 53, 56, 59]. Important factors in Flanders et al. were clear lines of authority in collaborative and effective multidisciplinary teams. Organisation readiness for change was perceived as both a barrier and a facilitator to research implementation but higher staff consensus was associated with higher engagement in organisational change [60]. Strategies in Dobbins et al. were thought to be more effective if they were implemented in organisations with learning culture and practices, or facilitated an organisational learning culture themselves, where Flanders et al. reported solutions to hospital safety problems often created more work or change from long-standing practices, which proved a barrier to overcome [53, 61]. Individual resistance to change in the form of process concerns led to higher levels of dissatisfaction [50].

#### Provide resources to support change

Individuals were conscious of the need for implementation strategies to be adequately resourced [48–50, 55, 56, 58, 59, 61]. There was anxiety in the study by Döpp et al. around promoting research implementation programs, due to the fear of receiving more referrals than could be handled with current resourcing [55]. Managers mention service pressures as a major barrier in changing practice, with implementation research involvement dependent on workload and other professional commitments [50, 56]. Lack of time prevented evidence reviews being performed, and varied access to human resources such as librarians were also identified as barriers [58, 59]. Policy-makers and managers appreciated links to expert researchers, especially those who had infrequent or irregular contact with the academic sector previously [49]. Managers typically viewed engagement with research implementation as a transactional idea, wanting funding for time release (beyond salary costs), while researchers and others from the academic sector consider knowledge exchange inherently valuable [48]. Vulnerability around leadership skills and knowledge in the study by Kitson et al. exposed the importance of training, education, and professional development opportunities. Ongoing training in critical appraisal of research literature was viewed as a predictor of whether systematic reviews influenced program planning [61].

#### Employ effective communication strategies

Studies and study participants expressed different preferences for the format and mode of contact for implementation strategies [48, 51, 52, 55, 56, 59, 64]. Face to face contact was preferred by the majority of participants in the study by Waqa et al. and was useful in acquiring and accessing relevant data or literature to inform the writing of policy briefs [51]. Telephone calls were perceived as successful in Döpp et al. because they increased involvement and opportunity to ask questions [55]. Electronic communication formats in the study by Bullock et al. provided examples of evidence-based knowledge transfer from academic settings to the clinical setting. Fellows spent time reading literature at the university and would then send that information to the clinical workplace in an email, while managers stated that the availability of website information positively influenced its use [48]. Regular contact in the form of reminders encouraged actions, with the study by Dagenais et al. finding lack of ongoing, regular contact with knowledge brokers in the field limitated research implementation programs [52].

#### Action change mechanism

Reviewers interpreted the domains (analytical themes) representing a model of implementation strategy success to lead to a change mechanism. Change mechanisms refer to the actions taken by study participants to implement research into practice. Studies did not explicitly measure the change mechanisms that lead to the implementation of research into practice. Instead, implicit measurements of change mechanisms were reported such as knowledge gain and intention to act measures.

## Discussion

This review found that there are numerous implementation strategies that can be utilised to promote evidence-informed policy and management decisions in healthcare. These relate to the ‘authority effect’ from a simple low-cost policy brief and knowledge improvement from a complex multifaceted workshop with ongoing technical assistance and distribution of instructional digital materials [46, 47]. The resource intensity of these strategies was relatively low. It was evident that providing more resource-intensive strategies is not always better than less, as the addition of a knowledge broker to a tailored targeted messaging strategy was less effective than the messages alone [9]. Due to the paucity of studies evaluating the effectiveness of implementation strategies, understanding why some implementation strategies succeed where others fail in different contexts is important for future strategy design. The thematic synthesis of the wider non-effectiveness literature included in our review has lead us to develop a model of implementation strategy design that may action a change mechanism for evidence-informed policy and management decisions in healthcare [48–61, 63, 64].

Our findings were concomitant with change management theories. The conceptual model of how themes interrelated both within and across studies includes similar stages to ‘Kotter’s 8 Step Change Model’ [65]. Leadership behaviours are commonly cited as organisational change drivers due to the formal power and authority that leaders have within organisations [66–68]. This supports the ‘authority effect’ described in Beynon et al. and the value decision-makers placed on information credited to experts they trust [46]. Authoritative messages are considered a key component of an effective policy brief, and therefore, organisations should consider partnering with authoritative institutions, research groups, or individuals to augment the legitimacy of their message when producing policy briefs [69]. Change management research proposes change-related training improves understanding, knowledge, and skills to embed a change vision at a group level [70–72]. The results of our review support this view that providing adequate training resources to decision-makers can improve understanding, knowledge, and skills, leading to desired change. The results of our thematic synthesis appear to support knowledge broker strategies in theory. Multi-component research implementation strategies are thought to have greater effects than simple strategies [73, 74]. However, the addition of knowledge brokers to a tailored targeted messaging research implementation strategy in Dobbins et al. was less effective than the messages alone [9]. This may indicate that in some cases, simple research implementation strategies may be more effective than complex, multi-component ones. Further development of strategies is needed to ensure that a number of different implementation options are available, which can be tailored to individual health contexts. A previous review by LaRocca et al. supports this finding, asserting that in some cases, complex strategies may diminish key messages and reduce understanding of information presented [10]. Further, the knowledge broker strategy in Dobbins et al. had little or no engagement from 30% of participants allocated to this group, emphasising the importance of tailoring strategy complexity and intensity to organisational need.

This systematic review was limited both in the quantity and quality of studies that met inclusion criteria. Previous reviews have been similarly limited in the paucity of high-quality research evaluating the effectiveness of research implementation strategies in the review context area [10, 29, 32, 75]. The limited number of retrieved experimental, quantitatively evaluated effectiveness studies, means the results of this review were mostly based on non-experimental qualitative data without an evaluation of effectiveness. Non-blinding of participants could have biased qualitative responses. Participants could have felt pressured to respond in a positive way if they did not wish to lose previously provided implementation resources, and responses could vary depending on the implementation context and what changes were being made, for example, if additional resources were being implemented to fill an existing evidence-to-practice gap, versus the disinvestment of resources due to a lack of supportive evidence. Despite these limitations, we believe our comprehensive search strategy retrieved a relatively complete identification of studies in the field of research. A previous Cochrane review in the same implementation context area recently identified only one study (also captured in our review) using their search strategy and inclusion criteria [33, 76]. A meta-analysis was unable to be performed due to the limited amount of studies and high levels of heterogeneity in study approaches, as such, the results of this synthesis should be interpreted with caution. However, synthesising data narratively and thematically allowed this review to examine not only the effectiveness of research implementation strategies in the context area but also the mechanisms behind inter-relating factors perceived to be associated with effective strategies. Since our original search strategy, we have been unable to identify additional full-texts from the 11 titles excluded due to no data reporting (e.g. protocol, abstract). However, the Developing and Evaluating Communication strategies to support Informed Decisions and practice based on Evidence (DECIDE) project has since developed a number of tools to improve the dissemination of evidence-based recommendations [77]. In addition, support for the relationship development, face to face interaction, and focus on organisational climates themes in our conceptual model is supported by the full version [78] of an excluded summary article [79], identified after the original search strategy.

Studies measured behaviour changes considered on the third level of the Kirkpatrick Hierarchy but did not measure whether those behaviour changes led to their intended improved societal outcomes (level 4, Kirkpatrick Hierarchy). Future research should also evaluate changes in health and organisational outcomes. The conceptualisation of factors perceived to be associated with effective strategies and the inter-relationship between these factors should be interpreted with caution as it was based on low levels of evidence according to the National Health and Medical Research Council (NHMRC) of Australia designations [80]. Therefore, there is a need for the association between these factors and effective strategies to be rigorously evaluated. Further conceptualisation of how to evaluate research implementation strategies should consider how to include health and organisation outcome measures to better understand how improved evidence-informed decision-making can lead to greater societal benefits. Future research should aim to improve the relatively low number of high-quality randomised controlled trials evaluating the effectiveness of research implementation strategies for promoting evidence-informed policy and management decisions in healthcare. This might allow formal meta-analysis to be performed, providing indications of what research implementation strategies are effective in which context.

## Conclusions

Evidence is developing to support the use of research implementation strategies for promoting evidence-informed policy and management decisions in healthcare. A number of inter-relating factors were thought to influence the effectiveness of strategies through establishing an imperative for change, building trust, developing a shared vision, and action change mechanisms. Employing effective communication strategies and providing resources to support change underpin these factors, which should inform the design of future implementation strategies.

## Additional files

Additional file 1: PRISMA 2009 checklist. (DOCX 26 kb)

Additional file 2: Search Strategy. (DOCX 171 kb)

Additional file 3: Data extraction 1 and 2. (XLSX 884 kb)

Additional file 4: Full list of 96 articles and reasons for full-text exclusion. (DOCX 125 kb)

## Abbreviations

CEO: Chief executive officer
NGO: Non-government organisation
NHMRC: National Health and Medical Research Council
PRISMA: Preferred Reporting Items for Systematic Reviews and Meta-Analysis
RCT: Randomised controlled trial
UK: United Kingdom
USA: United States of America
