# Supplementary material for: The effectiveness of research implementation strategies for promoting evidence-informed policy and management decisions in healthcare: a systematic review
Source: Implement Sci. 2017 Nov 14;12:132. doi: 10.1186/s13012-017-0662-0 (PMC5686806; doi:10.1186/s13012-017-0662-0)
Supplement: Supplementary file 2 — Search Strategy. (DOCX 171 kb) [file 13012_2017_662_MOESM2_ESM.docx]

**Additional file 2: Search Strategy**

Population 1 AND population 2 AND intervention

| **Population 1** | **Population 2** | **Intervention** |
| --- | --- | --- |
| Health* | Decision maker* | Knowledge translation OR knowledge transfer OR knowledge implementation OR knowledge utili?ation OR knowledge dissemination OR knowledge adoption OR knowledge change* OR knowledge evaluation OR knowledge use* OR knowledge institutionali?ation OR knowledge communication |
| Hospital* | Policy maker* | research translation OR research transfer OR research implementation OR research utili?ation OR research dissemination OR research adoption OR research change* OR research evaluation OR research use* OR research institutionali?ation OR research communication |
|  | Manager* | evidence translation OR evidence transfer OR evidence implementation OR evidence utili?ation OR evidence dissemination OR evidence adoption OR evidence change* OR evidence evaluation OR evidence use* OR evidence institutionali?ation OR evidence communication |
|  | Director* | Translation of knowledge OR translation of research OR translation of evidence OR transfer of knowledge OR transfer of research OR transfer of evidence OR systematic review evidence |
|  | Executive* |  |
|  | Leader* |  |
|  | Public health* |  |
|  | Health administrat* |  |
|  | Hospital administrat* |  |
|  | Health department* |  |
|  | Hospital department* |  |

**Scopus 2/02/2016**

| **1** | [( TITLE-ABS-KEY ( **health*** )  OR  TITLE-ABS-KEY ( **hospital*** ) )  AND  PUBYEAR  >  **1999**](http://www-scopus-com.ezproxy.lib.monash.edu.au/search/save/action.url?activity=allAction&userSearchID=46&origin=savedsearch) | **3518001** |
| --- | --- | --- |
| **2** | ( TITLE-ABS-KEY ( "decision maker*" ) OR TITLE-ABS-KEY ( "policy maker*" ) OR TITLE-ABS-KEY ( manager* ) OR TITLE-ABS-KEY ( director* ) OR TITLE-ABS-KEY ( executive* ) OR TITLE-ABS-KEY ( leader* ) OR TITLE-ABS-KEY ( "public health*" ) OR TITLE-ABS-KEY ( "health administ*" ) OR TITLE-ABS-KEY ( "hospital administ*" ) OR TITLE-ABS-KEY ( "health department*" ) OR TITLE-ABS-KEY ( "hospital department*" ) ) AND PUBYEAR > 1999 | **792837** |
| **3** | [( TITLE-ABS-KEY ( **"research translation"** )  OR  TITLE-ABS-KEY ( **"research transfer"** )  OR  TITLE-ABS-KEY ( **"research implementation"** )  OR  TITLE-ABS-KEY ( **"research utili?ation"** ) OR  TITLE-ABS-KEY ( **"research dissemination"** )  OR  TITLE-ABS-KEY ( **"research adoption"** )  OR  TITLE-ABS-KEY ( **"research change*"** )  OR  TITLE-ABS-KEY ( **"research evaluation"** )  OR  TITLE-ABS-KEY ( **"research use*"** )  OR  TITLE-ABS-KEY ( **"research institutionali?ation"** )  OR  TITLE-ABS-KEY ( **"research communication"** )  OR  TITLE-ABS-KEY ( **"Knowledge translation"** )  OR  TITLE-ABS-KEY ( **"Knowledge transfer"** )  OR TITLE-ABS-KEY ( **"knowledge implementation"** )  OR  TITLE-ABS-KEY ( **"knowledge utili?ation"** )  OR  TITLE-ABS-KEY ( **"knowledge dissemination"** )  OR  TITLE-ABS-KEY ( **"knowledge adoption"** ) OR  TITLE-ABS-KEY ( **"knowledge change*"** )  OR  TITLE-ABS-KEY ( **"knowledge evaluation"** )  OR  TITLE-ABS-KEY ( **"knowledge use*"** )  OR  TITLE-ABS-KEY ( **"knowledge institutionali?ation"** ) OR  TITLE-ABS-KEY ( **"knowledge communication"** )  OR  TITLE-ABS-KEY ( **"evidence translation"** )  OR  TITLE-ABS-KEY ( **"evidence transfer"** )  OR  TITLE-ABS-KEY ( **"evidence implementation"** )  OR TITLE-ABS-KEY ( **"evidence utili?ation"** )  OR  TITLE-ABS-KEY ( **"evidence dissemination"** )  OR  TITLE-ABS-KEY ( **"evidence adoption"** )  OR  TITLE-ABS-KEY ( **"evidence change*"** )  OR  TITLE-ABS-KEY ( **"evidence evaluation"** )  OR  TITLE-ABS-KEY ( **"evidence use*"** )  OR  TITLE-ABS-KEY ( **"evidence institutionali?ation"** )  OR TITLE-ABS-KEY ( **"evidence communication"** )  OR  TITLE-ABS-KEY ( **"translation of knowledge"** )  OR  TITLE-ABS-KEY ( **"translation of research"** )  OR  TITLE-ABS-KEY ( **"translation of evidence"** )  OR  TITLE-ABS-KEY ( **"transfer of knowledge"** )  OR TITLE-ABS-KEY ( **"transfer of research"** )  OR  TITLE-ABS-KEY ( **"translation of evidence"** ) OR  TITLE-ABS-KEY ( **"systematic review evidence"** )](http://www-scopus-com.ezproxy.lib.monash.edu.au/search/save/action.url?activity=allAction&userSearchID=50&origin=savedsearch) )  AND  PUBYEAR  >  **1999** | **30628** |
| **4** | ( ( TITLE-ABS-KEY ( **"research translation"** )  OR  TITLE-ABS-KEY ( **"research transfer"** )  OR  TITLE-ABS-KEY ( **"research implementation"** )  OR  TITLE-ABS-KEY ( **"research utili?ation"** )  OR  TITLE-ABS-KEY ( **"research dissemination"** )  OR  TITLE-ABS-KEY ( **"research adoption"** )  OR  TITLE-ABS-KEY ( **"research change*"** )  OR  TITLE-ABS-KEY ( **"research evaluation"** )  OR  TITLE-ABS-KEY ( **"research use*"** )  OR  TITLE-ABS-KEY ( **"research institutionali?ation"** )  OR  TITLE-ABS-KEY ( **"research communication"** )  OR  TITLE-ABS-KEY ( **"Knowledge translation"** )  OR  TITLE-ABS-KEY ( **"Knowledge transfer"** )  OR  TITLE-ABS-KEY ( **"knowledge implementation"** )  OR  TITLE-ABS-KEY ( **"knowledge utili?ation"** )  OR  TITLE-ABS-KEY ( **"knowledge dissemination"** )  OR  TITLE-ABS-KEY ( **"knowledge adoption"** ) OR  TITLE-ABS-KEY ( **"knowledge change*"** )  OR  TITLE-ABS-KEY ( **"knowledge evaluation"** )  OR  TITLE-ABS-KEY ( **"knowledge use*"** )  OR  TITLE-ABS-KEY ( **"knowledge institutionali?ation"** )  OR  TITLE-ABS-KEY ( **"knowledge communication"** )  OR  TITLE-ABS-KEY ( **"evidence translation"** )  OR  TITLE-ABS-KEY ( **"evidence transfer"** )  OR  TITLE-ABS-KEY ( **"evidence implementation"** )  OR  TITLE-ABS-KEY ( **"evidence utili?ation"** )  OR  TITLE-ABS-KEY ( **"evidence dissemination"** )  OR  TITLE-ABS-KEY ( **"evidence adoption"** )  OR  TITLE-ABS-KEY ( **"evidence change*"** )  OR  TITLE-ABS-KEY ( **"evidence evaluation"** )  OR  TITLE-ABS-KEY ( **"evidence use*"** )  OR  TITLE-ABS-KEY ( **"evidence institutionali?ation"** )  OR  TITLE-ABS-KEY ( **"evidence communication"** )  OR  TITLE-ABS-KEY ( **"translation of knowledge"** )  OR  TITLE-ABS-KEY ( **"translation of research"** )  OR  TITLE-ABS-KEY ( **"translation of evidence"** )  OR  TITLE-ABS-KEY ( **"transfer of knowledge"** )  OR  TITLE-ABS-KEY ( **"transfer of research"** )  OR  TITLE-ABS-KEY ( **"translation of evidence"** )  OR  TITLE-ABS-KEY ( **"systematic review evidence"** ) )  AND  PUBYEAR  >  **1999** )  AND  ( ( TITLE-ABS-KEY ( **"decision maker*"** )  OR  TITLE-ABS-KEY ( **"policy maker*"** )  OR  TITLE-ABS-KEY ( **manager*** )  OR TITLE-ABS-KEY ( **director*** )  OR  TITLE-ABS-KEY ( **executive*** )  OR  TITLE-ABS-KEY ( **leader*** )  OR  TITLE-ABS-KEY ( **"public health*"** )  OR  TITLE-ABS-KEY ( **"health administ*"** )  OR  TITLE-ABS-KEY ( **"hospital administ*"** )  OR  TITLE-ABS-KEY ( **"health department*"** )  OR  TITLE-ABS-KEY ( **"hospital department*"** ) )  AND  PUBYEAR >  **1999** )  AND  ( ( TITLE-ABS-KEY ( **health*** )  OR  TITLE-ABS-KEY ( **hospital*** ) )  AND  PUBYEAR  >  **1999** ) | **2201** |

**Ovid medline 2/02/2016**

| 1 | limit 1 to yr="2000 -Current" | 1600878 |
| --- | --- | --- |
| 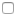 2 | (health* or hospital*).ab,kw,sh,ti. | 2802546 |
| 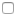 3 | ("decision maker*" or "policy maker*" or manager* or director* or executive* or leader* or "public health*" or "health administrat*" or "hospital administrat*" or "health department*" or "hospital department*").ab,kw,sh,ti. | 399921 |
| 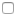 4 | ("Knowledge translation" or "knowledge transfer" or "knowledge implementation" or "knowledge utili?ation" or "knowledge dissemination" or "knowledge adoption" or "knowledge change*" or "knowledge evaluation" or "knowledge use*" or "knowledge institutionali?ation" or "knowledge communication" or "research translation" or "research transfer" or "research implementation" or "research utili?ation" or "research dissemination" or "research adoption" or "research change*" or "research evaluation" or "research use*" or "research institutionali?ation" or "research communication" or "evidence translation" or "evidence transfer" or "evidence implementation" or "evidence utili?ation" or "evidence dissemination" or "evidence adoption" or "evidence change*" or "evidence evaluation" or "evidence use*" or "evidence institutionali?ation" or "evidence communication" or "Translation of knowledge" or "translation of research" or "translation of evidence" or "transfer of knowledge" or "transfer of research" or "transfer of evidence" or "systematic review evidence").ab,kw,sh,ti. | 7669 |
| 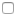 **5** | **1 and 2 and 3 and 4** | **1056** |

**Articles identified from publication list of experts in the field of implementation science**

| **Author** | **Qualification** | **Institution** | **Articles Identified** |
| --- | --- | --- | --- |
| Maureen Dobbins | B.Sc.N., Ph.D. | McMaster University | 0 |
| Penelope Fitzpatrick (Beynon) | Master of Arts (MA) | Institute of Development Studies | 1 [1] |
| Anita Kothari | PhD, MHSc, BSc | Western Health Sciences | 0 |
| Donna Ciliska | B.Sc.N., M.Sc.N., Ph.D. | McMaster University | 0 |
| Rebecca LaRocca | BScN, Masters of Science in Nursing | McMaster University | 0 |
| Leslea Peirson | PhD, Master’s degree, [Community Psychology](https://www.linkedin.com/edu/fos?id=100925&trk=prof-edu-field_of_study) | McMaster University | 0 |
| John Lavis | PhD | McMaster University | 2 [2, 3] |

**Articles identified from reference screen of included articles**

| **Reference** | **Citation ID** | **Author** | **Year** | **Title** | **Articles Identified** |
| --- | --- | --- | --- | --- | --- |
| [4] | 3813 | Brownson | 2007 | The Effect of Disseminating Evidence-Based Interventions That Promote Physical Activity to Health Departments | 4 [5-8] |
| [9] | 2580 | Bullock | 2012 | The Personal Touch: Exchanging Knowledge Through Manager Placements in Research Teams | 2 [10, 11] |
| [12] | 1156 | Campbell | 2011 | Evidence Check: knowledge brokering to commission research reviews for policy | 0 |
| [13] | 3819 | Chambers | 2012 | Use of evidence from systematic reviews to inform commissioning decisions: a case study | 0 |
| [14] | 2472 | Champagne | 2014 | Organizational impact of evidence-informed decision making training initiatives: a case study comparison of two approaches | 0 |
| [15] | 3642 | Courtney | 2007 | Using organizational assessment as a tool for program change | 0 |
| [16] | 503 | Dagenais | 2015 | Collaborative development and implementation of a knowledge brokering program to promote research use in Burkina Faso, West Africa | 0 |
| [17] | 1315 | Dobbins | 2001 | Factors of the innovation, organization, environment, and individual that predict the influence five systematic reviews had on public health decisions. | 1 [18] |
| [19] | 2873 | Dobbins | 2009 | A randomized controlled trial evaluating the impact of knowledge translation and exchange strategies | 0 |
| [20] | 761 | Dopp | 2013 | Determinants for the effectiveness of implementing an occupational therapy intervention in routine dementia care | o |
| [21] | 1551 | Flanders | 2009 | Hospitalists as Emerging Leaders in Patient Safety: Lessons Learned and Future Directions | 0 |
| [22] | 1349 | Gagliardi | 2008 | Fostering knowledge exchange between researchers and decision-makers: Exploring the effectiveness of a mixed-methods approach | 1 [23] |
| [24] | 470 | Kitson | 2011 | Clinical nursing leaders_, team members_ and service managers_ experiences of implementing evidence at a local level | 0 |
| [25] | 1676 | Uneke | 2015 | Implementation of a health policy advisory committee as a knowledge translation platform: the Nigeria experience | 0 |
| [26] | 3862 | Waqa | 2013 | Knowledge brokering between researchers and policymakers in Fiji to develop policies to reduce obesity: a process evaluation | 0 |
|  |  |  |  |  |  |

**Articles identified from systematic reviews**

| **Reference** | **Author** | **Title** | **Year** | **Journal** | **Articles Identified** |
| --- | --- | --- | --- | --- | --- |
| [27] | Moore | Translating Health Services Research into Practice in the Safety Net | 2016 | Health Services Research | 0 |
| [28] | Milat | Narrative review of models and success factors for scaling up public health interventions | 2015 | Implementation Science | 0 |
| [29] | Bornbaum | Exploring the function and effectiveness of knowledge brokers as facilitators of knowledge translation in health-related settings: a systematic review and thematic analysis | 2015 | Implementation Science | 9 [12, 30-37] |
| [38] | Abdullah | Measuring the Effectiveness of Mentoring as a Knowledge Translation Intervention for Implementing Empirical Evidence: A Systematic Review | 2014 | Worldviews on Evidence-Based Nursing | 0 |
| [39] | Archambault | Wikis and Collaborative Writing Applications in Health Care: A Scoping Review | 2013 | J Med Internet Res | 0 |
| [40] | Barac | Scoping review of toolkits as a knowledge translation strategy in health | 2014 | BMC Medical Informatics and Decision Making | 2 [41, 42] |
| [43] | Bostrom | What do we know about knowledge translation in the care of older adults? A scoping review | 2012 | J Am Med Dir Assocgul | 1 [44] |
| [45] | Chambers | The Seniors Health Research Transfer Network knowledge network model: system-wide implementation for health and healthcare of seniors | 2010 | Healthcare Management Forum | 3 [46-48] |
| [49] | Murthy | Interventions to improve the use of systematic reviews in decision-making by health system managers, policy makers and clinicians | 2012 | Cochrane Database Syst Rev | 8 [50-57] |
| [58] | Moore | What works to increase the use of research in population health policy and programmes: a review | 2011 | Evidence & Policy: A Journal of Research, Debate and Practice | 2 [59, 60] |
| [61] | Williamson | How Can the Use of Evidence in Mental Health Policy Be Increased? A Systematic Review | 2015 | Psychiatric Services | 9 [36, 62-69] |
| [70] | Thompson | Interventions aimed at increasing research use in nursing: a systematic review | 2007 | Implementation Science | 0 |
| [71] | Scott | Systematic review of knowledge translation strategies in the allied health professions | 2012 | Implement Sci | 0 |
| [72] | Ospina | A systematic review of the effectiveness of knowledge translation interventions for chronic noncancer pain management | 2013 | Pain Research and Management | 1 [73] |
| [74] | Orton | The use of research evidence in public health decision making processes: systematic review | 2011 | PLoS One | 0 |
| [75] | Oliver | New directions in evidence-based policy research: a critical analysis of the literature | 2014 | Health Res Policy Syst | 0 |
| [76] | Oliver | A systematic review of barriers to and facilitators of the use of evidence by policymakers | 2014 | BMC Health Services Research | 1 [13] |
| [77] | Noonan | Knowledge translation and implementation in spinal cord injury: a systematic review | 2014 | Spinal cord | 0 |
| [78] | McCormack | A realist review of interventions and strategies to promote evidence-informed healthcare: a focus on change agency | 2013 | Implement Sci | 0 |
| [79] | Gifford | Managerial leadership for nurses' use of research evidence: an integrative review of the literature | 2007 | Worldviews on Evidence‐Based Nursing | 2 [80, 81] |
| [82] | Elueze | Evaluating the effectiveness of knowledge brokering in health research: a systematised review with some bibliometric information | 2015 | Health Information & Libraries Journal | 5 [83-87] |
| [88] | LaRocca | The effectiveness of knowledge translation strategies used in public health: a systematic review. | 2012 | BMC public health | 3 [89-91] |
| [92] | Chambers | Maximizing the Impact of Systematic Reviews in Health Care Decision Making: A Systematic Scoping Review of Knowledge‐Translation Resources | 2011 | Milbank Quarterly | 5 [93-97] |
| [98] | Dagenais | Knowledge transfer on complex social interventions in public health: a scoping study | 2013 | PloS one | 6 [4, 99-103] |
| [104] | Bunn | Strategies to promote the impact of systematic reviews on healthcare policy: a systematic review of the literature | 2011 | Evidence & Policy: A Journal of Research, Debate and Practice | 0 |
| [57] | Perrier | Interventions encouraging the use of systematic reviews by health policymakers and managers: a systematic review | 2011 | Implementation Science | 1 [105] |

**References**

1. Beynon P, Chapoy C, Gaarder M, Masset E: **What difference does a policy brief make?**: Institute of Development Studies and 3ie; 2012.

2. Moat KA, Lavis JN, Clancy SJ, El-Jardali F, Pantoja T: **Evidence briefs and deliberative dialogues: perceptions and intentions to act on what was learnt**. *Bulletin of the World Health Organization* 2014, **92**(1):20-28.

3. Wilson MG, Grimshaw JM, Haynes RB, Hanna SE, Raina P, Gruen R, Ouimet M, Lavis JN: **A process evaluation accompanying an attempted randomized controlled trial of an evidence service for health system policymakers**. *Health research policy and systems* 2015, **13**(1):78.

4. Brownson RC, Ballew P, Brown KL, Elliott MB, Haire-Joshu D, Heath GW, Kreuter MW: **The effect of disseminating evidence-based interventions that promote physical activity to health departments**. *American journal of public health* 2007, **97**(10):1900-1907.

5. Brownson RC, Kreuter MW, Arrington BA, True WR: **Translating scientific discoveries into public health action: how can schools of public health move us forward?** *Public health reports* 2006:97-103.

6. Kerner J, Rimer B, Emmons K: **Introduction to the special section on dissemination: dissemination research and research dissemination: how can we close the gap?** *Health psychology : official journal of the Division of Health Psychology, American Psychological Association* 2005, **24**(5):443-446.

7. Oldenburg BF, Sallis JF, Ffrench ML, Owen N: **Health promotion research and the diffusion and institutionalization of interventions**. *Health education research* 1999, **14**(1):121-130.

8. Kerner JF, Guirguis-Blake J, Hennessy KD, Brounstein PJ, Vinson C, Schwartz RH, Myers BA, Briss P: **Translating research into improved outcomes in comprehensive cancer control**. *Cancer causes & control : CCC* 2005, **16 Suppl 1**:27-40.

9. Bullock A, Morris ZS, Atwell C: **Exchanging knowledge through healthcare manager placements in research teams**. *The Service Industries Journal* 2013, **33**(13-14):1363-1380.

10. Antil T, Desrochers M, Joubert P, Bouchard C: **Implementation of an innovative grant programme to build partnerships between researchers, decision-makers and practitioners: the experience of the Quebec Social Research Council**. *Journal of health services research & policy* 2003, **8 Suppl 2**:35-43.

11. Lockett A, El Enany N, Currie G, Oborn E, Barrett M, Racko G, Bishop S, Waring J: **A formative evaluation of Collaboration for Leadership in Applied Health Research and Care (CLAHRC): institutional entrepreneurship for service innovation**. 2014.

12. Campbell D, Donald B, Moore G, Frew D: **Evidence check: knowledge brokering to commission research reviews for policy**. *Evid Policy* 2011, **7**.

13. Chambers D, Grant R, Warren E, Pearson S-A, Wilson P: **Use of evidence from systematic reviews to inform commissioning decisions: a case study**. *Evidence & Policy: A Journal of Research, Debate and Practice* 2012, **8**(2):141-148.

14. Champagne F, Lemieux-Charles L, Duranceau M-F, MacKean G, Reay T: **Organizational impact of evidence-informed decision making training initiatives: a case study comparison of two approaches**. *Implementation Science* 2014, **9**(1):53.

15. Courtney KO, Joe GW, Rowan-Szal GA, Simpson DD: **Using organizational assessment as a tool for program change**. *Journal of Substance Abuse Treatment* 2007, **33**(2):131-137.

16. Dagenais C, Somé TD, Boileau-Falardeau M, McSween-Cadieux E, Ridde V: **Collaborative development and implementation of a knowledge brokering program to promote research use in Burkina Faso, West Africa**. *Global health action* 2015, **8**.

17. Dobbins M, Cockerill R, Barnsley J, Ciliska D: **Factors of the innovation, organization, environment, and individual that predict the influence five systematic reviews had on public health decisions**. *Int J Technol Assess Health Care* 2001, **17**.

18. Dobbins M, Cockerill R, Barnsley J: **Factors affecting the utilization of systematic reviews. A study of public health decision makers**. *Int J Technol Assess Health Care* 2001, **17**(2):203-214.

19. Dobbins M, Hanna SE, Ciliska D, Manske S, Cameron R, Mercer SL: **A randomized controlled trial evaluating the impact of knowledge translation and exchange strategies**. *Implement Sci* 2009, **4**.

20. Döpp CM, Graff MJ, Rikkert MGO, van der Sanden MWN, Vernooij-Dassen MJ: **Determinants for the effectiveness of implementing an occupational therapy intervention in routine dementia care**. *Implementation Science* 2013, **8**(1):1.

21. Flanders SA, Kaufman SR, Saint S, Parekh VI: **Hospitalists as emerging leaders in patient safety: lessons learned and future directions**. *Journal of patient safety* 2009, **5**(1):3-8.

22. Gagliardi AR, Fraser N, Wright FC, Lemieux-Charles L, Davis D: **Fostering knowledge exchange between researchers and decision-makers: exploring the effectiveness of a mixed-methods approach**. *Health Policy* 2008, **86**(1):53-63.

23. Ouimet M, Amara N, Landry R, Lavis J: **Direct interactions medical school faculty members have with professionals and managers working in public and private sector organizations: A cross-sectional study**. *Scientometrics* 2007, **72**(2):307-323.

24. Kitson A, Silverston H, Wiechula R, Zeitz K, Marcoionni D, Page T: **Clinical nursing leaders', team members' and service managers' experiences of implementing evidence at a local level**. *Journal of nursing management* 2011, **19**(4):542-555.

25. Uneke CJ, Ndukwe CD, Ezeoha AA, Uro-Chukwu HC, Ezeonu CT: **Implementation of a health policy advisory committee as a knowledge translation platform: the Nigeria experience**. *International Journal of Health Policy and Management* 2015, **4**(3):161-168.

26. Waqa G, Mavoa H, Snowdon W, Moodie M, Nadakuitavuki R, Mc Cabe M, Swinburn B: **Participants' perceptions of a knowledge-brokering strategy to facilitate evidence-informed policy-making in Fiji**. *BMC public health* 2013, **13**:725.

27. Moore SL, Fischer I, Havranek EP: **Translating Health Services Research into Practice in the Safety Net**. *Health Services Research* 2016, **51**(1):16-31.

28. Milat AJ, Bauman A, Redman S: **Narrative review of models and success factors for scaling up public health interventions**. *Implementation Science* 2015, **10**(1):1-11.

29. Bornbaum CC, Kornas K, Peirson L, Rosella LC: **Exploring the function and effectiveness of knowledge brokers as facilitators of knowledge translation in health-related settings: a systematic review and thematic analysis**. *Implementation Science* 2015, **10**(1):1-12.

30. Cameron D, Russell DJ, Rivard L, Darrah J, Palisano R: **Knowledge brokering in children’s rehabilitation organizations: perspectives from administrators**. *J Contin Educ Health Prof* 2011, **31**.

31. Robeson P, Dobbins M, DeCorby K: **Life as a knowledge broker in public health**. *J Can Health Libr Assoc* 2008, **29**.

32. van Kammen J, de Savigny D, Sewankambo N: **Using knowledge brokering to promote evidence-based policy-making: The need for support structures**. *Bull World Health Organ* 2006, **84**(8):608-612.

33. Van Kammen J, Jansen CW, Bonsel GJ, Kremer JA, Evers JL, Wladimiroff JW: **Technology assessment and knowledge brokering: the case of assisted reproduction in The Netherlands**. *International journal of technology assessment in health care* 2006, **22**(03):302-306.

34. Waqa G, Mavoa H, Snowdon W, Moodie M, Nadakuitavuki R, Mc Cabe M: **Participants’ perceptions of a knowledge-brokering strategy to facilitate evidence-informed policy-making in Fiji**. *BMC Public Health* 2013, **13**.

35. Waqa G, Mavoa H, Snowdon W, Moodie M, Schultz J, McCabe M: **Knowledge brokering between researchers and policymakers in Fiji to develop policies to reduce obesity: a process evaluation**. *Implement Sci* 2013, **8**.

36. Ward V, Smith S, Hamer S: **Exploring knowledge exchange: a useful framework for practice and policy**. *Soc Sci Med* 2012, **74**.

37. Phillips SJ: **PILOTING KNOWLEDGE BROKERS TO PROMOTE INTEGRATED STROKE CARE IN ATLANTIC CANADA**. *Evidence in action, acting on evidence*:57.

38. Abdullah G, Rossy D, Ploeg J, Davies B, Higuchi K, Sikora L, Stacey D: **Measuring the effectiveness of mentoring as a knowledge translation intervention for implementing empirical evidence: a systematic review**. *Worldviews Evid Based Nurs* 2014, **11**(5):284-300.

39. Archambault PM, van de Belt TH, Grajales III FJ, Faber MJ, Kuziemsky CE, Gagnon S, Bilodeau A, Rioux S, Nelen WL, Gagnon M-P *et al*: **Wikis and Collaborative Writing Applications in Health Care: A Scoping Review**. *J Med Internet Res* 2013, **15**(10):e210.

40. Barac R, Stein S, Bruce B, Barwick M: **Scoping review of toolkits as a knowledge translation strategy in health**. *BMC Medical Informatics and Decision Making* 2014, **14**(1):1-9.

41. Damschroder LJ, Lowery JC: **Evaluation of a large-scale weight management program using the consolidated framework for implementation research (CFIR)**. *Implementation Science* 2013, **8**(1):51.

42. Leape L, Rogers G, Hanna D, Griswold P, Federico F, Fenn C, Bates D, Kirle L, Clarridge B: **Developing and implementing new safe practices: voluntary adoption through statewide collaboratives**. *Quality and Safety in Health Care* 2006, **15**(4):289-295.

43. Bostrom AM, Slaughter SE, Chojecki D, Estabrooks CA: **What do we know about knowledge translation in the care of older adults? A scoping review**. *Journal of the American Medical Directors Association* 2012, **13**(3):210-219.

44. CHAMBERS R, KNIGHT F, CAMPBELL I: **A pilot study of the introduction of audit into nursing homes**. *Age and ageing* 1996, **25**(6):465-469.

45. Chambers LW, Luesby D, Brookman C, Harris M, Lusk E: **The Seniors Health Research Transfer Network knowledge network model: system-wide implementation for health and healthcare of seniors**. In: *Healthcare Management Forum: 2010*: Elsevier; 2010: 4-9.

46. Li LC, Grimshaw JM, Nielsen C, Judd M, Coyte PC, Graham ID: **Use of communities of practice in business and health care sectors: A systematic review**. *Implement Sci* 2009, **4**(27):16.

47. Graham ID, Tetroe J: **CIHR research: How to translate health research knowledge into effective healthcare action**. *Healthcare Quarterly* 2007, **10**(3).

48. Robeson P: **Networking in public health: Exploring the value of networks to the National Collaborating Centres for Public Health**: National Collaborating Centre for Methods and Tools; 2009.

49. Murthy L, Shepperd S, Clarke MJ, Garner SE, Lavis JN, Perrier L, Roberts NW, Straus SE: **Interventions to improve the use of systematic reviews in decision-making by health system managers, policy makers and clinicians**. *Cochrane Database Syst Rev* 2012, **9**.

50. Gülmezoglu A, Villar J, Grimshaw J, Piaggio G, Lumbiganon P, Langer A: **Cluster randomized trial of an active, multifaceted information dissemination intervention based on The WHO Reproductive health library to change obstetric practices: methods and design issues [ISRCTN14055385]**. *BMC medical research methodology* 2004, **4**(1):1.

51. Mason J, Freemantle N, Browning G: **Impact of Effective Health Care bulletin on treatment of persistent glue ear in children: time series analysis**. *BMJ* 2001, **323**(7321):1096-1097.

52. Rosenbaum SE, Glenton C, Oxman AD: **Summary-of-findings tables in Cochrane reviews improved understanding and rapid retrieval of key information**. *Journal of clinical epidemiology* 2010, **63**(6):620-626.

53. Booth A, Price C: **Models for disseminating information on clinical effectiveness in health organisations: lessons from the Sheffield DICE Project**. *Journal of Clinical Excellence* 2000, **1**(4):193-200.

54. Ciliska D, Hayward S, Dobbins M, Brunton G, Underwood J: **Transferring public-health nursing research to health-system planning: Assessing the relevance and accessibility of systematic reviews**. *Can J Nurs Res* 1999, **31**.

55. DeBeck K, Kerr T: **The use of knowledge translation and legal proceedings to support evidence-based drug policy in Canada: opportunities and ongoing challenges**. *Open Medicine* 2010, **4**(3):e167-e170.

56. Lavis JN, Wilson MG, Grimshaw JM, Haynes RB, Hanna S, Raina P, Gruen R, Ouimet M: **Effects of an evidence service on health-system policy makers' use of research evidence: a protocol for a randomised controlled trial**. *Implement Sci* 2011, **6**:51.

57. Perrier L, Mrklas K, Lavis JN, Straus SE: **Interventions encouraging the use of systematic reviews by health policymakers and managers: a systematic review**. *Implement Sci* 2011, **6**:43.

58. Moore G, Redman S, Haines M, Todd A: **What works to increase the use of research in population health policy and programmes: a review**. *Evidence & Policy: A Journal of Research, Debate and Practice* 2011, **7**(3):277-305.

59. Taylor RS, Reeves BC, Ewings PE, Taylor RJ: **Critical appraisal skills training for health care professionals: a randomized controlled trial [ISRCTN46272378]**. *BMC medical education* 2004, **4**(1):30.

60. Denis JL, Lomas J, Stipich N: **Creating receptor capacity for research in the health system: the Executive Training for Research Application (EXTRA) program in Canada**. *Journal of health services research & policy* 2008, **13 Suppl 1**:1-7.

61. Williamson A, Makkar SR, McGrath C, Redman S: **How Can the Use of Evidence in Mental Health Policy Be Increased? A Systematic Review**. *Psychiatric Services* 2015.

62. Glisson C, Hemmelgarn A, Green P, Williams NJ: **Randomized trial of the Availability, Responsiveness and Continuity (ARC) organizational intervention for improving youth outcomes in community mental health programs**. *Journal of the American Academy of Child and Adolescent Psychiatry* 2013, **52**(5):493-500.

63. Saldana L, Chamberlain P: **Supporting implementation: the role of community development teams to build infrastructure**. *American journal of community psychology* 2012, **50**(3-4):334-346.

64. Chamberlain P, Roberts R, Jones H, Marsenich L, Sosna T, Price JM: **Three collaborative models for scaling up evidence-based practices**. *Administration and policy in mental health* 2012, **39**(4):278-290.

65. Driedger SM, Kothari A, Graham ID, Cooper E, Crighton EJ, Zahab M, Morrison J, Sawada M: **If you build it, they still may not come: outcomes and process of implementing a community-based integrated knowledge translation mapping innovation**. *Implementation Science* 2010, **5**(1):1-13.

66. Luck J, Hagigi F, Parker LE, Yano EM, Rubenstein LV, Kirchner JE: **A social marketing approach to implementing evidence-based practice in VHA QUERI: the TIDES depression collaborative care model**. *Implement Sci* 2009, **4**:64.

67. McGrath PJ, Lingley-Pottie P, Emberly DJ, Thurston C, McLean C: **Integrated Knowledge Translation in Mental Health: Family Help as an Example**. *Journal of the Canadian Academy of Child and Adolescent Psychiatry* 2009, **18**(1):30-37.

68. Stark C, Innes A, Szymczynska P, Forrest L, Proctor K: **Dementia knowledge transfer project in a rural area**. *Rural and remote health* 2013, **13**(2):2060.

69. Feinberg ME, Jones D, Greenberg MT, Osgood DW, Bontempo D: **Effects of the Communities That Care Model in Pennsylvania on Change in Adolescent Risk and Problem Behaviors**. *Prevention science : the official journal of the Society for Prevention Research* 2010, **11**(2):163-171.

70. Thompson DS, Estabrooks CA, Scott-Findlay S, Moore K, Wallin L: **Interventions aimed at increasing research use in nursing: A systematic review**. *Implementation Science* 2007, **2**(1):1-16.

71. Scott SD, Albrecht L, O'Leary K, Ball GD, Hartling L, Hofmeyer A, Jones CA, Klassen TP, Kovacs Burns K, Newton AS *et al*: **Systematic review of knowledge translation strategies in the allied health professions**. *Implementation science : IS* 2012, **7**:70.

72. Ospina MB, Taenzer P, Rashiq S, MacDermid JC, Carr E, Chojecki D, Harstall C, Henry JL: **A systematic review of the effectiveness of knowledge translation interventions for chronic noncancer pain management**. *Pain research & management : the journal of the Canadian Pain Society = journal de la societe canadienne pour le traitement de la douleur* 2013, **18**(6):e129-141.

73. Ferguson F, Holdsworth L, Rafferty D: **A national framework for supporting improvements in the physiotherapy assessment and management of low back pain: the Scottish experience**. *Physiotherapy* 2010, **96**(3):198-205.

74. Orton L, Lloyd-Williams F, Taylor-Robinson D, O'Flaherty M, Capewell S: **The use of research evidence in public health decision making processes: systematic review**. *PLoS One* 2011, **6**(7):e21704.

75. Oliver K, Lorenc T, Innvær S: **New directions in evidence-based policy research: a critical analysis of the literature**. *Health Res Policy Syst* 2014, **12**(1):34.

76. Oliver K, Innvar S, Lorenc T, Woodman J, Thomas J: **A systematic review of barriers to and facilitators of the use of evidence by policymakers**. *BMC Health Services Research* 2014, **14**(1):1-12.

77. Noonan V, Wolfe D, Thorogood N, Park S, Hsieh J, Eng J: **Knowledge translation and implementation in spinal cord injury: a systematic review**. *Spinal cord* 2014, **52**(8):578-587.

78. McCormack B, Rycroft-Malone J, DeCorby K, Hutchinson AM, Bucknall T, Kent B, Schultz A, Snelgrove-Clarke E, Stetler C, Titler M: **A realist review of interventions and strategies to promote evidence-informed healthcare: a focus on change agency**. *Implement Sci* 2013, **8**(1):107.

79. Gifford W, Davies B, Edwards N, Griffin P, Lybanon V: **Managerial leadership for nurses' use of research evidence: an integrative review of the literature**. *Worldviews on Evidence‐Based Nursing* 2007, **4**(3):126-145.

80. Rutledge DN, Donaldson NE: **Building organizational capacity to engage in research utilization**. *The Journal of nursing administration* 1995, **25**(10):12-16.

81. Hodnett ED, Kaufman K, O'Brien-Pallas L, Chipman M, Watson-MacDonell J, Hunsburger W: **A strategy to promote research-based nursing care: Effects on childbirth outcomes**. *Research in Nursing & Health* 1996, **19**(1):13-20.

82. Elueze IN: **Evaluating the effectiveness of knowledge brokering in health research: a systematised review with some bibliometric information**. *Health Information & Libraries Journal* 2015, **32**(3):168-181.

83. Michaels S: **Matching knowledge brokering strategies to environmental policy problems and settings**. *Environmental Science & Policy* 2009, **12**(7):994-1011.

84. Van Kammen J, de Savigny D, Sewankambo N: **Using knowledge brokering to promote evidence-based policy-making: the need for support structures**. *Bulletin of the World Health Organization* 2006, **84**(8):608-612.

85. Wehrens R, Bekker M, Bal R: **The construction of evidence-based local health policy through partnerships: Research infrastructure, process, and context in the Rotterdam 'Healthy in the City' programme**. *Journal of public health policy* 2010, **31**(4):447-460.

86. Hamel N, Schrecker T: **Unpacking capacity to utilize research: A tale of the Burkina Faso public health association**. *Social science & medicine (1982)* 2011, **72**(1):31-38.

87. Frank J, Frost H, Geddes R, Haw S, Jackson C, Jepson R: **Experiences of knowledge brokering for evidence-informed public health, policy, and practice: 3 years of the Scottish Collaboration for Public Health Research and Policy**. *Lancet* 2012, **380**.

88. LaRocca R, Yost J, Dobbins M, Ciliska D, Butt M: **The effectiveness of knowledge translation strategies used in public health: a systematic review**. *BMC public health* 2012, **12**(1):751.

89. Barwick MA, Peters J, Boydell K: **Getting to Uptake: Do Communities of Practice Support the Implementation of Evidence-Based Practice?** *Journal of the Canadian Academy of Child and Adolescent Psychiatry* 2009, **18**(1):16-29.

90. Forsetlund L, Bradley P, Forsen L, Nordheim L, Jamtvedt G, Bjorndal A: **Randomised controlled trial of a theoretically grounded tailored intervention to diffuse evidence-based public health practice**. In: *BMC Medical Education.* edn.; 2003.

91. Hanbury A, Wallace L, Clark M: **Use of a time series design to test effectiveness of a theory-based intervention targeting adherence of health professionals to a clinical guideline**. *British journal of health psychology* 2009, **14**(Pt 3):505-518.

92. Chambers D, Wilson PM, Thompson CA, Hanbury A, Farley K, Light K: **Maximizing the impact of systematic reviews in health care decision making: A systematic scoping review of knowledge-translation resources**. *Milbank Quarterly* 2011, **89**(1):131-156.

93. Handoll H, Madhok R: **Utility of the Cochrane Database of Systematic Reviews for evidence-based health policy and practice: a case study**. *Journal of Clinical Excellence* 2001, **3**(2):59-68.

94. Packer C, Hyde C: **Does providing timely access to and advice on existing reviews of research influence health authority purchasing**. *Public Health Medicine* 2000, **2**:20-24.

95. Rashiq S, Barton P, Harstall C, Schopflocher D, Taenzer P: **The Alberta Ambassador Program: delivering Health Technology Assessment results to rural practitioners**. *BMC medical education* 2006, **6**:21.

96. Thornton-Jones H, Hampshaw S, Soltani H: **Evidence, policy making and the art of the possible: a methodology applied to screening**. *Journal of Clinical Excellence* 2002, **4**(1):9-16.

97. Chambers D, Grant R, Warren E, Pearson S-A, Wilson P: **Use of systematic review evidence to inform local decision-making in the National Health Service: a case study of eating disorders**. In: *Joint Cochrane and Campbell Colloquium October: 2010*; 2010: 22.

98. Dagenais C, Malo M, Robert E, Ouimet M, Berthelette D, Ridde V: **Knowledge transfer on complex social interventions in public health: a scoping study**. *PloS one* 2013, **8**(12):e80233.

99. Adily A, Black D, Graham ID, Ward JE: **Research engagement and outcomes in public health and health services research in Australia**. *Australian and New Zealand journal of public health* 2009, **33**(3):258-261.

100. Kelly JA, Somlai AM, DiFranceisco WJ, Otto-Salaj LL, McAuliffe TL, Hackl KL, Heckman TG, Holtgrave DR, Rompa D: **Bridging the gap between the science and service of HIV prevention: transferring effective research-based HIV prevention interventions to community AIDS service providers**. *Am J Public Health* 2000, **90**(7):1082-1088.

101. Klein JD, Allan MJ, Elster AB, Stevens D, Cox C, Hedberg VA, Goodman RA: **Improving adolescent preventive care in community health centers**. *Pediatrics* 2001, **107**(2):318-327.

102. Masuda JR, Robinson K, Elliott S, Eyles J: **Disseminating chronic disease prevention "to or with" Canadian public health systems**. *Health education & behavior : the official publication of the Society for Public Health Education* 2009, **36**(6):1026-1050.

103. McCormick L, Tompkins NO: **Diffusion of CDC's Guidelines to Prevent Tobacco Use and Addiction**. *The Journal of school health* 1998, **68**(2):43-45.

104. Bunn F, Sworn K: **Strategies to promote the impact of systematic reviews on healthcare policy: a systematic review of the literature**. *Evidence & Policy: A Journal of Research, Debate and Practice* 2011, **7**(4):403-428.

105. Ciliska D, Hayward S, Dobbins M, Brunton G, Underwood J: **Transferring public-health nursing research to health-system planning: assessing the relevance and accessibility of systematic reviews**. *The Canadian journal of nursing research = Revue canadienne de recherche en sciences infirmieres* 1999, **31**(1):23-36.
