# Supplementary material for: The effectiveness of research implementation strategies for promoting evidence-informed policy and management decisions in healthcare: a systematic review
Source: Implement Sci. 2017 Nov 14;12:132. doi: 10.1186/s13012-017-0662-0 (PMC5686806; doi:10.1186/s13012-017-0662-0)
Supplement: Supplementary file 4 — Full list of 96 articles and reasons for full-text exclusion. (DOCX 125 kb) [file 13012_2017_662_MOESM4_ESM.docx]

**Additional file 4: Full List of 96 Articles and Reasons for Full-Text Exclusion**

| **Study details** | **Excluded on** | | **Reason** |
| --- | --- | --- | --- |
| #3468  Translating Health Services Research into Practice in the Safety Net  Health Services Research 2016;51(1):16-31 16p  Malden, Massachusetts Wiley-Blackwell 2016  [View Abstract & IDs](javascript:void(0))[Hide full text](javascript:void(0);)   \| **REFERENCE URL:** \| \| \| --- \| --- \| \|  \| [Change](javascript:void(0)) \| \| **UPLOADED FILES:** \| \| \| [moore 2016.pdf](https://regroup-production.s3.amazonaws.com/documents/ReviewReference/12061630/moore%202016.pdf?AWSAccessKeyId=AKIAIH6DLCYZGRZ772MQ&Expires=1495782876&Signature=XfMVCHYZ%2FAc%2FxU1%2B6U17XNMv4Pg%3D) \| Primary PDF \|   [Add another PDF](javascript:void(0))  [View historyAdd a note](javascript:void(0);)[Move study to Full text review](javascript:void(0)) | 28/06/16 | | Review article |
| #2330  Narrative review of models and success factors for scaling up public health interventions  Implementation Science 2015;10(1):1-11 11p  BioMed Central 2015  [View Abstract & IDs](javascript:void(0))[Hide full text](javascript:void(0);)   \| **REFERENCE URL:** \| \| \| --- \| --- \| \|  \| [Change](javascript:void(0)) \| \| **UPLOADED FILES:** \| \| \| [milat 2015.pdf](https://regroup-production.s3.amazonaws.com/documents/ReviewReference/12060492/milat%202015.pdf?AWSAccessKeyId=AKIAIH6DLCYZGRZ772MQ&Expires=1495782900&Signature=j10P6OuS%2Bceo8Kn%2FjpHjjzFiCEE%3D) \| Primary PDF \|   [Add another PDF](javascript:void(0))  [View historyView 1 note](javascript:void(0);)[Move study to Full text review](javascript:void(0)) | 28/06/16 | | Review article |
|  |  | |  |
| #1275  Exploring the function and effectiveness of knowledge brokers as facilitators of knowledge translation in health-related settings: a systematic review and thematic analysis  Implementation Science 2015;16():1-12 12p  BioMed Central 2015  [View Abstract & IDs](javascript:void(0))[Add full text](javascript:void(0);)  [View historyView 1 note](javascript:void(0);)[Move study to Full text review](javascript:void(0)) | 28/06/16 | | Review article |
|  |  | |  |
| #2245 - Abdullah 2014  Abdullah, Ghadah; Rossy, Dianne; Ploeg, Jenny; Davies, Barbara; Higuchi, Kathryn; Sikora, Lindsey; Stacey, Dawn  Measuring the Effectiveness of Mentoring as a Knowledge Translation Intervention for Implementing Empirical Evidence: A Systematic Review  Worldviews on Evidence-Based Nursing 2014;11(5):284-300 17p  Malden, Massachusetts Wiley-Blackwell 2014  [View Abstract & IDs](javascript:void(0))[View full text](javascript:void(0);)  [View historyView 1 note](javascript:void(0);)[Move study to Full text review](javascript:void(0)) | 28/06/16 | | Review article |
|  |  | |  |
| #1579 - Ahuja 2012  Ahuja, T.; Taylor, S.; Dossa, A.; Vosilla, A.; Pang, W.; Mira, A.; Jennings, S.  How collaboration can bring about change: Effective knowledge translation on self-monitoring of blood glucose  Canadian Journal of Diabetes October 2012;1)():S4  2012 October  [View Abstract & IDs](javascript:void(0))[View full text](javascript:void(0);)  [View historyAdd a note](javascript:void(0);)[Move study to Full text review](javascript:void(0)) | 28/06/16 | | No data reported (e.g. protocol paper, abstract, ect.) |
|  |  | |  |
| #3240 - Alamri 2015  Alamri, Sultan H.; Kennedy, Courtney C.; Marr, Sharon; Lohfeld, Lynne; Skidmore, Carly J.; Papaioannou, Alexandra  Strategies to overcome barriers to implementing osteoporosis and fracture prevention guidelines in long-term care: a qualitative analysis of action plans suggested by front line staff in Ontario, Canada  BMC Geriatrics Aug 1 2015;15():  2015 Aug 1  [View Abstract & IDs](javascript:void(0))[View full text](javascript:void(0);)  [View historyAdd a note](javascript:void(0);)[Move study to Full text review](javascript:void(0)) | 28/06/16 | | Does not examine the effect of a research implementation strategy on decision-making by healthcare policy-makers or managers |
|  |  | |  |
| #3795 - Archambault 2013  Archambault, Patrick M.; van de Belt, Tom H.; Grajales, Francisco J., III; Faber, Marjan J.; Kuziemsky, Craig E.; Gagnon, Susie; Bilodeau, Andrea; Rioux, Simon; Nelen, Willianne L.; Gagnon, Marie-Pierre; Turgeon, Alexis F.; Aubin, Karine; Gold, Irving; Poitras, Julien; Eysenbach, Gunther; Kremer, Jan A.; Legare, France  Wikis and collaborative writing applications in health care: A scoping review  Journal of Medical Internet Research Oct 2013;15(10):4-38  2013 Oct  [View Abstract & IDs](javascript:void(0))[View full text](javascript:void(0);)  [View historyView 1 note](javascript:void(0);)[Move study to Full text review](javascript:void(0)) | 28/06/16 | | Review article |
|  |  | |  |
| #1255 - Armstrong 2011  Armstrong, Rebecca; Swinburn, Boyd; Moore, Laurence; Dobbins, Maureen; Anderson, Laurie; Petticrew, Mark; Clark, Rachel; Conning, Rebecca; Moodie, Marj; Carter, Robert  An exploratory cluster randomised controlled trial of knowledge translation strategies to support evidence-informed decision-making in local governments (The KT4LG study).  BMC Public Health 2011;11(100968562):  2011  [View Abstract & IDs](javascript:void(0))[View full text](javascript:void(0);)  [View historyAdd a note](javascript:void(0);)[Move study to Full text review](javascript:void(0)) | 28/06/16 | | No data reported (e.g. protocol paper, abstract, ect.) |
|  |  | |  |
| #3118 - Barac 2014  Barac, Raluca; Stein, Sherry; Bruce, Beth; Barwick, Melanie  Scoping review of toolkits as a knowledge translation strategy in health.  BMC medical informatics and decision making 2014;14():121  2014  [View Abstract & IDs](javascript:void(0))[Add full text](javascript:void(0);)  [View historyAdd a note](javascript:void(0);)[Move study to Full text review](javascript:void(0)) | 28/06/16 | | Review article |
|  |  | |  |
| #1653 - Bennett 2013  Bennett, S.; Paina, L.; Ssengooba, F.; Waswa, D.; M'Imunya, J. M.  The impact of Fogarty International Center research training programs on public health policy and program development in Kenya and Uganda  BMC Public Health 2013;13(1):  2013  [View Abstract & IDs](javascript:void(0))[View full text](javascript:void(0);)  [View historyAdd a note](javascript:void(0);)[Move study to Full text review](javascript:void(0)) | 28/06/16 | | Strategy not delivered to a healthcare policy-maker or manager |
|  |  | |  |
| #2899 - Black 2002  Black, N.; Hutchings, A.  Reduction in the use of surgery for glue ear: did national guidelines have an impact?  Quality & Safety in Health Care 2002;11():121-4  2002  [View Abstract & IDs](javascript:void(0))[View full text](javascript:void(0);)  [View historyAdd a note](javascript:void(0);)[Move study to Full text review](javascript:void(0)) | 28/06/16 | | Strategy not delivered to a healthcare policy-maker or manager |
|  |  | |  |
| #1277 - Bornbaum 2015  Bornbaum, C. C.; Kornas, K.; Peirson, L.; Rosella, L. C.  Exploring the function and effectiveness of knowledge brokers as facilitators of knowledge translation in health-related settings: a systematic review and thematic analysis  Implement Sci 2015;10(1):162  2015  [View Abstract & IDs](javascript:void(0))[Add full text](javascript:void(0);)  [View historyAdd a note](javascript:void(0);)[Move study to Full text review](javascript:void(0)) | 28/06/16 | | Review article |
|  |  | |  |
| #3729 - Bostrom 2012  Bostrom, A. M.; Slaughter, S. E.; Chojecki, D.; Estabrooks, C. A.  What Do We Know About Knowledge Translation in the Care of Older Adults? A Scoping Review  Journal of the American Medical Directors Association March 2012;13(3):210-219  2012 March  [View Abstract & IDs](javascript:void(0))[Add full text](javascript:void(0);)  [View historyAdd a note](javascript:void(0);)[Move study to Full text review](javascript:void(0)) | 28/06/16 | | Review article |
|  |  | |  |
| #3730 - Boström 2012  Boström, A. M.; Slaughter, S. E.; Chojecki, D.; Estabrooks, C. A.  What Do We Know About Knowledge Translation in the Care of Older Adults? A Scoping Review  Journal of the American Medical Directors Association 2012;13(3):210-219  2012  [View Abstract & IDs](javascript:void(0))[View full text](javascript:void(0);)  [View historyAdd a note](javascript:void(0);)[Move study to Full text review](javascript:void(0)) | 28/06/16 | | Review article |
|  |  | |  |
| #1756 - Boulet 2008  Boulet, L. P.  Improving knowledge transfer on chronic respiratory diseases: a Canadian perspective. How to translate recent advances in respiratory diseases into day-to-day care  Dec 2008;12(10):758S-763S  2008 Dec  [View Abstract & IDs](javascript:void(0))[View full text](javascript:void(0);)  [View historyAdd a note](javascript:void(0);)[Move study to Full text review](javascript:void(0)) | 04/07/16 | | Does not examine the effect of a research implementation strategy on decision-making by healthcare policy-makers or managers |
|  |  | |  |
| #2229 - Bowen 2006  Bowen, S.  Marginalized evidence: Effective knowledge translation strategies for low awareness issues  Healthcare Management Forum 2006;19(3):38-44  2006  [View Abstract & IDs](javascript:void(0))[View full text](javascript:void(0);)  [View historyView 1 note](javascript:void(0);)[Move study to Full text review](javascript:void(0)) | 11/07/16 | | Does not examine the effect of a research implementation strategy on decision-making by healthcare policy-makers or managers |
|  |  | |  |
| #1913 - Bregman 2009  Bregman, D.; Korman, A.; Shetach, A.; Shalom, N.  An internet-based simulation system for training and development of regional-healthcare-centers managers  Studies in Health Technology & Informatics 2009;150():789-93  2009  [View Abstract & IDs](javascript:void(0))[View full text](javascript:void(0);)  [View historyAdd a note](javascript:void(0);)[Move study to Full text review](javascript:void(0)) | 04/07/16 | | Does not measure a relevant outcome based on the Kirkpatrick hierarchy evaluation model |
|  |  | |  |
| #3269 - Brigham 2013  Brigham, L. L.  A study of how health visitors exchange knowledge in the context of organisational and policy change  Knowledge Management 2013;12(1):17-31  2013  [View Abstract & IDs](javascript:void(0))[View full text](javascript:void(0);)  [View historyView 1 note](javascript:void(0);)[Move study to Full text review](javascript:void(0)) | 11/07/16 | | Duplicate publication/data |
|  |  | |  |
| #494 - Bullock 2012  Bullock, A.; Morris, Z. S.; Atwell, C.  Collaboration between health services managers and researchers: making a difference?  Journal of health services research & policy Apr 2012;17():2-10  2012 Apr  [View Abstract & IDs](javascript:void(0))[Add full text](javascript:void(0);)  [View historyAdd a note](javascript:void(0);)[Move study to Full text review](javascript:void(0)) | 11/07/16 | | Duplicate publication/data |
|  |  | |  |
| #3244 - Bunn 2011  Bunn, Frances; Sworn, Katie  Strategies to promote the impact of systematic reviews on healthcare policy: a systematic review of the literature  Nov 2011;7(4):403-428  2011 Nov  [View Abstract & IDs](javascript:void(0))[Add full text](javascript:void(0);)  [View historyAdd a note](javascript:void(0);)[Move study to Full text review](javascript:void(0)) | 28/06/16 | | Review article |
|  |  | |  |
| #3275 - Caldwell 2012  Caldwell, S. E. M.; Mays, N.  Studying policy implementation using a macro, meso and micro frame analysis: the case of the Collaboration for Leadership in Applied Health Research & Care (CLAHRC) programme nationally and in North West London  Health Research Policy and Systems 2012;10():  2012  [View Abstract & IDs](javascript:void(0))[View full text](javascript:void(0);)  [View historyAdd a note](javascript:void(0);)[Move study to Full text review](javascript:void(0)) | 11/07/16 | | Does not examine the effect of a research implementation strategy on decision-making by healthcare policy-makers or managers |
|  |  | |  |
| #3608 - Chambers 2010  Chambers, D.; Grant, R.; Warren, E.; Pearson, S. A.; Wilson, P.  Use of systematic review evidence to inform local decision-making in the National Health Service: a case study of eating disorders. Poster presentation at the Joint Cochrane and Campbell Colloquium; 2010 Oct 18-22; Keystone, Colorado, USA [abstract]  Cochrane Database of Systematic Reviews, Supplement 2010;Suppl(Cd000002):63  2010  [View Abstract & IDs](javascript:void(0))[View full text](javascript:void(0);)  [View historyView 1 note](javascript:void(0);)[Move study to Full text review](javascript:void(0)) | 25/07/16 | | not a peer-reviewed publication (posters etc) |
|  |  | |  |
| #3818 - Chambers 2010  Chambers, Duncan; Grant, Rod; Warren, Erica; Pearson, Sally-Anne; Wilson, Paul  Use of systematic review evidence to inform local decision-making in the National Health Service: a case study of eating disorders  2010;18():22  2010  [View Abstract & IDs](javascript:void(0))[View full text](javascript:void(0);)  [View historyAdd a note](javascript:void(0);)[Move study to Full text review](javascript:void(0)) | 11/07/16 | | Duplicate publication/data |
|  |  | |  |
| #2234 - Chambers 2011  Chambers, D.; Wilson, P. M.; Thompson, C. A.; Hanbury, A.; Farley, K.; Light, K.  Maximizing the impact of systematic reviews in health care decision making: A systematic scoping review of knowledge-translation resources  Milbank Quarterly March 2011;89(1):131-156  2011 March  [View Abstract & IDs](javascript:void(0))[Add full text](javascript:void(0);)  [View historyAdd a note](javascript:void(0);)[Move study to Full text review](javascript:void(0)) | 04/07/16 | | Review article |
|  |  | |  |
| #2472 - Champagne 2014  Champagne F.; Lemieux-Charles L.; Duranceau MF.; MacKean G.; Reay T.  Organizational impact of evidence-informed decision making training initiatives: a case study comparison of two approaches.  Implementation science : IS 2014;9():53  2014  [View Abstract & IDs](javascript:void(0))[View full text](javascript:void(0);)  [View historyAdd a note](javascript:void(0);)[Move study to Full text review](javascript:void(0)) | 27/10/16 | | Duplicate publication/data |
|  |  | |  |
| #95 - Colantonio 2008  Colantonio, A.; Kontos, P. C.; Gilbert, J. E.; Rossiter, K.; Gray, J.; Keightley, M. L.  After the crash: research-based theater for knowledge transfer  The Journal of continuing education in the health professions 2008;28(3):180-185  United States 2008  [View Abstract & IDs](javascript:void(0))[View full text](javascript:void(0);)  [View historyAdd a note](javascript:void(0);)[Move study to Full text review](javascript:void(0)) | 04/07/16 | | Strategy not delivered to a healthcare policy-maker or manager |
|  |  | |  |
| #3021 - Cooke 2013  Cooke, M.; Walker, R.  Research, transformational leadership and knowledge translation: A successful formula  Scandinavian Journal of Caring Sciences 2013;27(1):1-2  2013  [View Abstract & IDs](javascript:void(0))[View full text](javascript:void(0);)  [View historyAdd a note](javascript:void(0);)[Move study to Full text review](javascript:void(0)) | 04/07/16 | | Does not examine the effect of a research implementation strategy on decision-making by healthcare policy-makers or managers |
|  |  | |  |
| #1458 - Cumpston 2004  Cumpston, M.; Clark, K.; Spithoff, D.; Ohlsson, A.  The good news: evidence of the dissemination and influence of Cochrane systematic reviews [abstract]  12th Cochrane Colloquium: Bridging the Gaps; 2004 Oct 2-6; Ottawa, Ontario, Canada 2004;():51-52  2004  [View Abstract & IDs](javascript:void(0))[Add full text](javascript:void(0);)  [View historyAdd a note](javascript:void(0);)[Move study to Full text review](javascript:void(0)) | 11/07/16 | | No data reported (e.g. protocol paper, abstract, ect.) |
|  |  | |  |
| #1109 - Dagenais 2013  Dagenais C.; Queuille L.; Ridde V.  Evaluation of a knowledge transfer strategy from a user fee exemption program for vulnerable populations in Burkina Faso.  Global health promotion Mar 2013;20(1 Suppl):70-9  2013 Mar  [View Abstract & IDs](javascript:void(0))[View full text](javascript:void(0);)  [View historyAdd a note](javascript:void(0);)[Move study to Full text review](javascript:void(0)) | 11/07/16 | | Not specific to evidence-based decision-making |
|  |  | |  |
| #504 - Dagenais 2015  Dagenais, C.; Some, T. D.; Boileau-Falardeau, M.; McSween-Cadieux, E.; Ridde, V.  Collaborative development and implementation of a knowledge brokering program to promote research use in Burkina Faso, West Africa  Glob Health Action 2015;8():26004  2015  [View Abstract & IDs](javascript:void(0))[View full text](javascript:void(0);)  [View historyView 1 note](javascript:void(0);)[Move study to Full text review](javascript:void(0)) | 14/08/16 | | Duplicate publication/data |
|  |  | |  |
| #2268 - Davies 2004  Davies, B.; Dobbins, M.; Edwards, N.; Griffin, P.; Ploeg, J.; Skelly, J.; Virani, T.  Methods for assessing the sustained use of research evidence in practice [abstract]  12th Cochrane Colloquium: Bridging the Gaps; 2004 Oct 2-6; Ottawa, Ontario, Canada 2004;():121-122  2004  [View Abstract & IDs](javascript:void(0))[View full text](javascript:void(0);)  [View historyAdd a note](javascript:void(0);)[Move study to Full text review](javascript:void(0)) | 11/07/16 | | No data reported (e.g. protocol paper, abstract, ect.) |
|  |  | |  |
| #3443 - D 1999  D., Ciliska; S., Hayward; M., Dobbins; G., Brunton; J., Underwood  Transferring public-health nursing research to health-system planning: assessing the relevance and accessibility of systematic reviews.  The Canadian journal of nursing research = Revue canadienne de recherche en sciences infirmières 1999;31():23-36  1999  [View Abstract & IDs](javascript:void(0))[View full text](javascript:void(0);)  [View historyAdd a note](javascript:void(0);)[Move study to Full text review](javascript:void(0)) | 04/07/16 | | Does not examine the effect of a research implementation strategy on decision-making by healthcare policy-makers or managers |
|  |  | |  |
| #3823 - DeBeck 2010  DeBeck, Kora; Kerr, Thomas  The use of knowledge translation and legal proceedings to support evidence-based drug policy in Canada: opportunities and ongoing challenges  Open Medicine 09/21 03/26/received 04/15/rev-request 06/17/revised 06/27/accepted 2010;4(3):e167-e170  Open Medicine Publications, Inc. 2010 09/21 03/26/received 04/15/rev-request 06/17/revised 06/27/accepted  [View Abstract & IDs](javascript:void(0))[View full text](javascript:void(0);)  [View historyAdd a note](javascript:void(0);)[Move study to Full text review](javascript:void(0)) | 04/07/16 | | Does not examine the effect of a research implementation strategy on decision-making by healthcare policy-makers or managers |
|  |  | |  |
| #2027 - Dobbins 2010  Dobbins M.; DeCorby K.; Robeson P.; Husson H.; Tirilis D.; Greco L.  A knowledge management tool for public health: health-evidence.ca.  BMC public health 2010;10():496  2010  [View Abstract & IDs](javascript:void(0))[View full text](javascript:void(0);)  [View historyAdd a note](javascript:void(0);)[Move study to Full text review](javascript:void(0)) | 11/07/16 | | Does not examine the effect of a research implementation strategy on decision-making by healthcare policy-makers or managers |
|  |  | |  |
| #83 - Donaldson 2008  Donaldson, N.; Rutledge, D.; Geiser, K.; Henriksen, K.; Battles, J. B.; Keyes, M. A.; Grady, M. L.  Advances in Patient Safety Role of the External Coach in Advancing Research Translation in Hospital-Based: Performance Improvement  2008;():  Rockville (MD) Agency for Healthcare Research and Quality (US) 2008  [View Abstract & IDs](javascript:void(0))[View full text](javascript:void(0);)  [View historyView 1 note](javascript:void(0);)[Move study to Full text review](javascript:void(0)) | 14/08/16 | | Strategy not delivered to a healthcare policy-maker or manager |
|  |  | |  |
| #395 - El-Jardali 2014  El-Jardali, F.; Lavis, J.; Moat, K.; Pantoja, T.; Ataya, N.  Capturing lessons learned from evidence-to-policy initiatives through structured reflection  Health Research Policy & Systems 2014;12():2  2014  [View Abstract & IDs](javascript:void(0))[View full text](javascript:void(0);)  [View historyAdd a note](javascript:void(0);)[Move study to Full text review](javascript:void(0)) | 07/07/16 | | Does not examine the effect of a research implementation strategy on decision-making by healthcare policy-makers or managers |
|  |  | |  |
| #1509 - Ellen 2014  Ellen, M. E.; Lavis, J. N.; Wilson, M. G.; Grimshaw, J.; Haynes, R. B.; Ouimet, M.; Raina, P.; Gruen, R.  Health system decision makers' feedback on summaries and tools supporting the use of systematic reviews: A qualitative study  Evidence and Policy 2014;10(3):337-359  2014  [View Abstract & IDs](javascript:void(0))[View full text](javascript:void(0);)  [View historyAdd a note](javascript:void(0);)[Move study to Full text review](javascript:void(0)) | 07/07/16 | | Does not examine the effect of a research implementation strategy on decision-making by healthcare policy-makers or managers |
|  |  | |  |
| #1094 - Elueze 2015  Elueze, I. N.  Evaluating the effectiveness of knowledge brokering in health research: a systematised review with some bibliometric information  Health Information & Libraries Journal Sep 2015;32(3):168-81  2015 Sep  [View Abstract & IDs](javascript:void(0))[Add full text](javascript:void(0);)  [View historyAdd a note](javascript:void(0);)[Move study to Full text review](javascript:void(0)) | 04/07/16 | | Review article |
|  |  | |  |
| #3290 - Evans 2014  Evans, Sarah; Scarbrough, Harry  Supporting knowledge translation through collaborative translational research initiatives: 'Bridging' versus 'blurring' boundary-spanning approaches in the UK CLAHRC initiative  Social Science & Medicine Apr 2014;106():119-127  2014 Apr  [View Abstract & IDs](javascript:void(0))[View full text](javascript:void(0);)  [View historyAdd a note](javascript:void(0);)[Move study to Full text review](javascript:void(0)) | 15/08/16 | | Does not measure a relevant outcome based on the Kirkpatrick hierarchy evaluation model |
|  |  | |  |
| #2879 - Falzon 2001  Falzon, L.; Booth, A.  REALISE-ing their potential?: implementing local library projects to support evidence-based health care...Research Evaluation to Audit Library and Information Support for EBHC  Health Information & Libraries Journal 2001;18(2):65-74 10p  Malden, Massachusetts Wiley-Blackwell 2001  [View Abstract & IDs](javascript:void(0))[View full text](javascript:void(0);)  [View historyAdd a note](javascript:void(0);)[Move study to Full text review](javascript:void(0)) | 11/07/16 | | Duplicate publication/data |
|  |  | |  |
| #2880 - Falzon 2001  Falzon, L.; Booth, A.  REALISE-ing their potential? Implementing local library projects to support evidence-based health care  Health Information and Libraries Journal Jun 2001 2001;18(2):65-74  2001 Jun 2001  [View Abstract & IDs](javascript:void(0))[View full text](javascript:void(0);)  [View historyAdd a note](javascript:void(0);)[Move study to Full text review](javascript:void(0)) | 11/07/16 | | Duplicate publication/data |
|  |  | |  |
| #57 - Finley 2008  Finley, G. A.; Forgeron, P.; Arnaout, M.  Action Research: Developing a Pediatric Cancer Pain Program in Jordan  Journal of Pain and Symptom Management 2008;35(4):447-454  2008  [View Abstract & IDs](javascript:void(0))[View full text](javascript:void(0);)  [View historyAdd a note](javascript:void(0);)[Move study to Full text review](javascript:void(0)) | 06/07/16 | | Strategy not delivered to a healthcare policy-maker or manager |
|  |  | |  |
| #3829  Frank, John; Frost, Helen; Geddes, Rosemary; Haw, Sally; Jackson, Caroline; Jepson, Ruth; McAteer, John; Mooney, John  Experiences of knowledge brokering for evidence-informed public health, policy, and practice: 3 years of the Scottish Collaboration for Public Health Research and Policy  The Lancet ;380():S39  Elsevier  [View Abstract & IDs](javascript:void(0))[View full text](javascript:void(0);)  [View historyView 1 note](javascript:void(0);)[Move study to Full text review](javascript:void(0)) | 11/07/16 | | No data reported (e.g. protocol paper, abstract, ect.) |
|  |  | |  |
| #393 - Gerrish 2014  Gerrish, Kate; Piercy, Hilary  Capacity Development for Knowledge Translation: Evaluation of an Experiential Approach through Secondment Opportunities  Worldviews on Evidence-Based Nursing 2014;11(3):209-216 8p  Malden, Massachusetts Wiley-Blackwell 2014  [View Abstract & IDs](javascript:void(0))[View full text](javascript:void(0);)  [View historyAdd a note](javascript:void(0);)[Move study to Full text review](javascript:void(0)) | 15/08/16 | | Strategy not delivered to a healthcare policy-maker or manager |
|  |  | |  |
| #2474 - Goldstine 2013  Goldstine, I.; Arratoon, C.; Buckley, N.; Deshpande, A.; Robeson, P.  Organizational impact of the canadian guideline for safe and effective use of opioids for chronic non-cancer pain: Survey on opioid policy  Pain Research and Management March-April 2013;18 (2)():e36  2013 March-April  [View Abstract & IDs](javascript:void(0))[View full text](javascript:void(0);)  [View historyView 1 note](javascript:void(0);)[Move study to Full text review](javascript:void(0)) | 06/07/16 | | No data reported (e.g. protocol paper, abstract, ect.) |
|  |  | |  |
| #406 - Hitch 2014  Hitch, Danielle; Rowan, Susan; Nicola-Richmond, Kelli  A case study of knowledge brokerage in occupational therapy  International Journal of Therapy & Rehabilitation 2014;21(8):389-396  2014  [View Abstract & IDs](javascript:void(0))[View full text](javascript:void(0);)  [View historyAdd a note](javascript:void(0);)[Move study to Full text review](javascript:void(0)) | 11/07/16 | | Does not examine the effect of a research implementation strategy on decision-making by healthcare policy-makers or managers |
|  |  | |  |
| #1671 - Kelly 2012  Kelly, A. M.; Pannifex, J.; Cetiner, E.  Implementation and impact of clinical network managed evidence-based practice implementation projects in Victorian emergency departments  Academic Emergency Medicine June 2012;19 (6)():745-746  2012 June  [View Abstract & IDs](javascript:void(0))[View full text](javascript:void(0);)  [View historyView 1 note](javascript:void(0);)[Move study to Full text review](javascript:void(0)) | 14/08/16 | | No data reported (e.g. protocol paper, abstract, ect.) |
|  |  | |  |
| #2327 - Kislov 2011  Kislov, R.  Multiprofessional communities of practice in a large-scale healthcare collaboration: Formation, identity building and knowledge sharing  Proceedings of the European Conference on Knowledge Management, ECKM 2011;2():1041-1048  2011  [View Abstract & IDs](javascript:void(0))[View full text](javascript:void(0);)  [View historyAdd a note](javascript:void(0);)[Move study to Full text review](javascript:void(0)) | 07/07/16 | | No data reported (e.g. protocol paper, abstract, ect.) |
|  |  | |  |
| #973 - LaRocca 2012  LaRocca R.; Yost J.; Dobbins M.; Ciliska D.; Butt M.  The effectiveness of knowledge translation strategies used in public health: a systematic review.  BMC public health 2012;12():751  2012  [View Abstract & IDs](javascript:void(0))[Add full text](javascript:void(0);)  [View historyAdd a note](javascript:void(0);)[Move study to Full text review](javascript:void(0)) | 04/07/16 | | Review article |
|  |  | |  |
| #2815 - Mays 2013  Mays, G. P.; Hogg, R. A.; Castellanos-Cruz, D. M.; Hoover, A. G.; Fowler, L. C.  Public health research implementation and translation: evidence from practice-based research networks  American Journal of Preventive Medicine Dec 2013;45(6):752-62  2013 Dec  [View Abstract & IDs](javascript:void(0))[View full text](javascript:void(0);)  [View historyAdd a note](javascript:void(0);)[Move study to Full text review](javascript:void(0)) | 15/08/16 | | Not specific to evidence-based decision-making |
| #2883 - McCormack 2013  McCormack, B.; Rycroft-Malone, J.; Decorby, K.; Hutchinson, A. M.; Bucknall, T.; Kent, B.; Schultz, A.; Snelgrove-Clarke, E.; Stetler, C.; Titler, M.; Wallin, L.; Wilson, V.  A realist review of interventions and strategies to promote evidence-informed healthcare: a focus on change agency  Implement Sci 2013;8():107  2013  [View Abstract & IDs](javascript:void(0))[View full text](javascript:void(0);)  [View historyAdd a note](javascript:void(0);)[Move study to Full text review](javascript:void(0)) | | 04/07/16 | Review article |
| #796 - Morris 2013  Morris, Zoe Slote; Bullock, Alison; Atwell, Christine  Developing engagement, linkage and exchange between health services managers and researchers: Experience from the UK  Journal of health services research & policy Apr 2013;18():23-29  2013 Apr  [View Abstract & IDs](javascript:void(0))[View full text](javascript:void(0);)  [View historyAdd a note](javascript:void(0);)[Move study to Full text review](javascript:void(0)) | | 15/08/16 | Not specific to evidence-based decision-making |
|  | |  |  |
| #2016 - Murnaghan 2013  Murnaghan D.; Morrison W.; Griffith EJ.; Bell BL.; Duffley LA.; McGarry K.; Manske S.  Knowledge exchange systems for youth health and chronic disease prevention: a tri-provincial case study.  Chronic diseases and injuries in Canada Sep 2013;33(4):257-66  2013 Sep  [View Abstract & IDs](javascript:void(0))[View full text](javascript:void(0);)  [View historyAdd a note](javascript:void(0);)[Move study to Full text review](javascript:void(0)) | | 25/07/16 | Strategy not delivered to a healthcare policy-maker or manager |
|  | |  |  |
| #1933 - Murthy 2012  Murthy, Lakshmi; Shepperd, Sasha; Clarke Mike, J.; Garner Sarah, E.; Lavis John, N.; Perrier, Laure; Roberts Nia, W.; Straus Sharon, E.  Interventions to improve the use of systematic reviews in decision-making by health system managers, policy makers and clinicians  Cochrane Database of Systematic Reviews 2012;(9):  2012  [View Abstract & IDs](javascript:void(0))[View full text](javascript:void(0);)  [View historyAdd a note](javascript:void(0);)[Move study to Full text review](javascript:void(0)) | | 06/07/16 | Review article |
|  | |  |  |
| #1138 - O'Connor 2015  O'Connor, Patricia; Fearfull, Anne  Evaluation of the Scottish Patient Safety Fellowship programme 2008-2013  Clinical Risk 2015;21(2/3):22-30 9p  Sage Publications, Ltd. 2015  [View Abstract & IDs](javascript:void(0))[View full text](javascript:void(0);)  [View historyAdd a note](javascript:void(0);)[Move study to Full text review](javascript:void(0)) | | 16/08/16 | Strategy not delivered to a healthcare policy-maker or manager |
|  | |  |  |
| #3604 - Orton 2011  Orton, L.; Lloyd-Williams, F.; Taylor-Robinson, D.; O'Flaherty, M.; Capewell, S.  The use of research evidence in public health decision making processes: Systematic review  PLoS ONE 2011;6(7):  2011  [View Abstract & IDs](javascript:void(0))[Add full text](javascript:void(0);)  [View historyAdd a note](javascript:void(0);)[Move study to Full text review](javascript:void(0)) | | 04/07/16 | Review article |
|  | |  |  |
| #3334 - Ospina 2013  Ospina, M. B.; Taenzer, P.; Rashiq, S.; MacDermid, J. C.; Carr, E.; Chojecki, D.; Harstall, C.; Henry, J. L.  A systematic review of the effectiveness of knowledge translation interventions for chronic noncancer pain management (Provisional abstract)  Pain Research and Management 2013;18(6):e129-141  2013  [View Abstract & IDs](javascript:void(0))[View full text](javascript:void(0);)  [View historyView 1 note](javascript:void(0);)[Move study to Full text review](javascript:void(0)) | | 04/07/16 | Review article |
|  | |  |  |
| #3635 - Pettman 2013  Pettman, T. L.; Armstrong, R.; Pollard, B.; Evans, R.; Stirrat, A.; Scott, I.; Davies-Jackson, G.; Waters, E.  Using evidence in health promotion in local government: contextual realities and opportunities  Health Promot J Austr 2013;24(1):72-5  2013  [View Abstract & IDs](javascript:void(0))[View full text](javascript:void(0);)  [View historyAdd a note](javascript:void(0);)[Move study to Full text review](javascript:void(0)) | | 06/07/16 | Does not examine the effect of a research implementation strategy on decision-making by healthcare policy-makers or managers |
|  | |  |  |
| #3850  Phillips, Stephen J  PILOTING KNOWLEDGE BROKERS TO PROMOTE INTEGRATED STROKE CARE IN ATLANTIC CANADA  Evidence in action, acting on evidence ;():57  [View Abstract & IDs](javascript:void(0))[View full text](javascript:void(0);)  [View historyView 1 note](javascript:void(0);)[Move study to Full text review](javascript:void(0)) | | 16/08/16 | No data reported (e.g. protocol paper, abstract, ect.) |
|  | |  |  |
| #2480 - Pinochet 2011  Pinochet, L. H. C.  An organizational view on formulating information security policies in hospitals  Mundo da Saude 2011;35(3):278-289  2011  [View Abstract & IDs](javascript:void(0))[Add full text](javascript:void(0);)  [View historyAdd a note](javascript:void(0);)[Move study to Full text review](javascript:void(0)) | | 14/08/16 | Does not examine the effect of a research implementation strategy on decision-making by healthcare policy-makers or managers |
|  | |  |  |
| #3853 - Robeson 2008  Robeson, Paula; Dobbins, Maureen; DeCorby, Kara  Life as a knowledge broker in public health  Journal of the Canadian Health Libraries Association/Journal de l'Association des bibliothèques de la santé du Canada 2008;29(3):79-82  2008  [View Abstract & IDs](javascript:void(0))[View full text](javascript:void(0);)  [View historyAdd a note](javascript:void(0);)[Move study to Full text review](javascript:void(0)) | | 07/07/16 | Does not examine the effect of a research implementation strategy on decision-making by healthcare policy-makers or managers |
|  | |  |  |
| #138 - Rosen 2000  Rosen R.  Applying research to health care policy and practice: medical and managerial views on effectiveness and the role of research.  Journal of health services research & policy Apr 2000;5(2):103-8  2000 Apr  [View Abstract & IDs](javascript:void(0))[View full text](javascript:void(0);)  [View historyAdd a note](javascript:void(0);)[Move study to Full text review](javascript:void(0)) | | 06/07/16 | Does not examine the effect of a research implementation strategy on decision-making by healthcare policy-makers or managers |
|  | |  |  |
| #3000 - Rushmer 2015  Rushmer, R. K.; Cheetham, M.; Cox, L.; Crosland, A.; Gray, J.; Hughes, L.; Hunter, D. J.; McCabe, K.; Seaman, P.; Tannahill, C.; Graff, P.  Research utilisation and knowledge mobilisation in the commissioning and joint planning of public health interventions to reduce alcohol-related harms: a qualitative case design using a cocreation approach (Structured abstract)  Health Technology Assessment Database 2015;(1):  Health Services and Delivery Research 2015  [View Abstract & IDs](javascript:void(0))[View full text](javascript:void(0);)  [View historyAdd a note](javascript:void(0);)[Move study to Full text review](javascript:void(0)) | | 06/07/16 | Does not examine the effect of a research implementation strategy on decision-making by healthcare policy-makers or managers |
|  | |  |  |
| #3420 - Salway 2013  Salway, S.; Turner, D.; Mir, G.; Bostan, B.; Carter, L.; Skinner, J.; Gerrish, K.; Ellison, G.  Towards equitable commissioning for our multiethnic society: a mixed-methods qualitative investigation of evidence utilisation by strategic commissioners and public health managers (Structured abstract)  Health Technology Assessment Database 2013;(1):  Health Services and Delivery Research 2013  [View Abstract & IDs](javascript:void(0))[View full text](javascript:void(0);)  [View historyAdd a note](javascript:void(0);)[Move study to Full text review](javascript:void(0)) | | 06/07/16 | Does not examine the effect of a research implementation strategy on decision-making by healthcare policy-makers or managers |
|  | |  |  |
| #3404 - Saul 2013  Saul, Jessie E.; Willis, Cameron D.; Bitz, Jennifer; Best, Allan  A time-responsive tool for informing policy making: rapid realist review  Implementation Science 2013;8():103  2013  [View Abstract & IDs](javascript:void(0))[View full text](javascript:void(0);)  [View historyAdd a note](javascript:void(0);)[Move study to Full text review](javascript:void(0)) | | 04/07/16 | Does not examine the effect of a research implementation strategy on decision-making by healthcare policy-makers or managers |
|  | |  |  |
| #732 - Stark 2013  Stark, C.; Innes, A.; Szymczynska, P.; Forrest, L.; Proctor, K.  Dementia knowledge transfer project in a rural area  Rural And Remote Health 2013;13(2):2060-2060  Australia Deakin University 2013  [View Abstract & IDs](javascript:void(0))[View full text](javascript:void(0);)  [View historyAdd a note](javascript:void(0);)[Move study to Full text review](javascript:void(0)) | | 11/07/16 | Does not examine the effect of a research implementation strategy on decision-making by healthcare policy-makers or managers |
|  | |  |  |
| #55 - Sullivan 2013  Sullivan, E.; Hegney, D. G.; Francis, K.  An action research approach to practice, service and legislative change  2013;21((Sullivan E., sullivan@dpar.com.au) Department of Nursing and Midwifery, Monash University, Melbourne, Australia):8-13  2013  [View Abstract & IDs](javascript:void(0))[View full text](javascript:void(0);)  [View historyAdd a note](javascript:void(0);)[Move study to Full text review](javascript:void(0)) | | 06/07/16 | Does not examine the effect of a research implementation strategy on decision-making by healthcare policy-makers or managers |
|  | |  |  |
| #3858 - Taylor 2004  Taylor, R. S.; Reeves, B. C.; Ewings, P. E.; Taylor, R. J.  Critical appraisal skills training for health care professionals: a randomized controlled trial [ISRCTN46272378]  BMC Med Educ Dec 7 2004;4(1):30  2004 Dec 7  [View Abstract & IDs](javascript:void(0))[View full text](javascript:void(0);)  [View historyView 1 note](javascript:void(0);)[Move study to Full text review](javascript:void(0)) | | 06/07/16 | Strategy not delivered to a healthcare policy-maker or manager |
|  | |  |  |
| #296 - Thamlikitkul 2006  Thamlikitkul, V.  Bridging the gap between knowledge and action for health: Case studies  Bulletin of the World Health Organization 2006;84(8):603-607  2006  [View Abstract & IDs](javascript:void(0))[View full text](javascript:void(0);)  [View historyAdd a note](javascript:void(0);)[Move study to Full text review](javascript:void(0)) | | 11/07/16 | Does not examine the effect of a research implementation strategy on decision-making by healthcare policy-makers or managers |
|  | |  |  |
| #1323 - Tomm-Bonde 2013  Tomm-Bonde,L.; Schreiber,R.S.; Allan,D.E.; MacDonald,M.; Pauly,B.; Hancock,T.  Fading vision: knowledge translation in the implementation of a public health policy intervention  Implementation Science 2013 2013;8():  2013 2013  [View Abstract & IDs](javascript:void(0))[View full text](javascript:void(0);)  [View historyView 1 note](javascript:void(0);)[Move study to Full text review](javascript:void(0)) | | 11/07/16 | Intervention is not a research implementation strategy |
|  | |  |  |
| #789 - Treweek 2013  Treweek, S.; Oxman, A. D.; Alderson, P.; Bossuyt, P. M.; Brandt, L.; Brozek, J.; Davoli, M.; Flottorp, S.; Harbour, R.; Hill, S.; Liberati, A.; Liira, H.; Schunemann, H. J.; Rosenbaum, S.; Thornton, J.; Vandvik, P. O.; Alonso-Coello, P.; Decide Consortium  Developing and Evaluating Communication Strategies to Support Informed Decisions and Practice Based on Evidence (DECIDE): protocol and preliminary results  Implementation Science 2013;8():6  2013  [View Abstract & IDs](javascript:void(0))[View full text](javascript:void(0);)  [View historyView 1 note](javascript:void(0);)[Move study to Full text review](javascript:void(0)) | | 06/07/16 | No data reported (e.g. protocol paper, abstract, ect.) |
|  | |  |  |
| #1677 - Uneke 2015  Uneke, C. J.; Ezeoha, A. E.; Uro-Chukwu, H. C.; Ezeonu, C. T.  Implementation of a mentorship programme on knowledge translation/management to improve policymakers' capacity for evidence-informed policymaking for the control of infectious diseases of poverty in Nigeria  Tropical Medicine and International Health September 2015;20():141  2015 September  [View Abstract & IDs](javascript:void(0))[View full text](javascript:void(0);)  [View historyAdd a note](javascript:void(0);)[Move study to Full text review](javascript:void(0)) | | 05/10/16 | not a peer-reviewed publication (posters etc) |
|  | |  |  |
| #1374 - Vaandrager 2010  Vaandrager, L.; Klerkx, L.; Naaldenberg, J.; Mareeuw, F. V.; de Regt, W.; Zandvliet, J.; Molleman, G.  From knowledge translation and knowledge brokerage towards knowledge co-creation: an innovation systems perspective on the public health knowledge infrastructure in The Netherlands  European Journal of Public Health Nov 2010;20():234-234  2010 Nov  [View Abstract & IDs](javascript:void(0))[View full text](javascript:void(0);)  [View historyAdd a note](javascript:void(0);)[Move study to Full text review](javascript:void(0)) | | 11/07/16 | No data reported (e.g. protocol paper, abstract, ect.) |
|  | |  |  |
| #1221 - Yousefi-Nooraie 2015  Yousefi-Nooraie, Reza; Dobbins, Maureen; Marin, Alexandra; Hanneman, Robert; Lohfeld, Lynne  The evolution of social networks through the implementation of evidence-informed decision-making interventions: a longitudinal analysis of three public health units in Canada  Implementation Science Dec 3 2015;10():  2015 Dec 3  [View Abstract & IDs](javascript:void(0))[View full text](javascript:void(0);)  [View historyAdd a note](javascript:void(0);)[Move study to Full text review](javascript:void(0)) | | 25/07/16 | Does not examine the effect of a research implementation strategy on decision-making by healthcare policy-makers or managers |
|  | |  |  |
| #3848  Does providing timely access and advice on existing reviews of research influence health authority purchasing  Public Health Medicine 2000;2():20-24 2000  [View Abstract & IDs](javascript:void(0))[Hide full text](javascript:void(0);)   \| **REFERENCE URL:** \| \| \| --- \| --- \| \|  \| [Change](javascript:void(0)) \| \| **UPLOADED FILES:** \| \| \|  \|  \|   [Add another PDF](javascript:void(0))  [View historyAdd a note](javascript:void(0);)[Move study to Full text review](javascript:void(0)) | | 25/07/2016 | Full-text unavailable |
|  | |  |  |
| Evidence briefs and deliberative dialogues: perceptions and intentions to act on what was learnt  Kaelan A Moat,a John N Lavis,b Sarah J Clancy,c Fadi El-Jardalid & Tomas Pantojae for the Knowledge Translation Platform Evaluation study team  *Bull World Health Organ* 2014;92:20–28 doi: http://dx.doi.org/10.2471/BLT.12.116806 | | Included | - |
|  | |  |  |
| A process evaluation accompanying an  attempted randomized controlled trial of  an evidence service for health system  policymakers  Michael G Wilson1,2,3*, Jeremy M Grimshaw4,5, R Brian Haynes3,6, Steven E Hanna3,8,9,10, Parminder Raina3,7,  Russell Gruen8, Mathieu Ouimet9,10 and John N Lavis1,2,3,11,12  Health Research Policy and Systems (2015) 13:78  DOI 10.1186/s12961-015-0066-z | | Included | - |
|  | |  |  |
| #3878 - Beynon 2012  Beynon, Penelope; Chapoy, Christelle; Gaarder, Marie; Masset, Edoardo  What difference does a policy brief make  2012;():  2012 | | Included | - |
|  | |  |  |
| **#3813 - Brownson 2007**  Brownson, Ross C.; Ballew, Paula; Brown, Kathrin L.; Elliott, Michael B.; Haire-Joshu, Debra; Heath, Gregory W.; Kreuter, Matthew W.  **The Effect of Disseminating Evidence-Based Interventions That Promote Physical Activity to Health Departments**  American Journal of Public Health 08/29/accepted 2007;97(10):1900-1907  American Public Health Association 2007 08/29/accepted | | Included | - |
|  | |  |  |
| **#2580 - Bullock 2012**  Bullock, A.; Morrisand, Z. S.; Atwell, C.  **The Personal Touch: Exchanging Knowledge Through Manager Placements in Research Teams**  Proceedings of the 13th European Conference on Knowledge Management, Vols 1 and 2 2012;():144-151  2012 | | Included | - |
|  | |  |  |
| **#1156 - Campbell 2011**  Campbell, D.; Donald, B.; Moore, G.; Frew, D.  **Evidence check: Knowledge brokering to commission research reviews for policy**  Evidence and Policy 2011;7(1):97-107  2011 | | Included | - |
|  | |  |  |
| **#3819 - Chambers 2012**  Chambers, Duncan; Grant, Rod; Warren, Erica; Pearson, Sally-Anne; Wilson, Paul  **Use of evidence from systematic reviews to inform commissioning decisions: a case study**  Evidence & Policy: A Journal of Research, Debate and Practice 2012;8(2):141-148  2012 | | Included | - |
|  | |  |  |
| **#2473 - Champagne 2014**  Champagne F.; Lemieux-Charles L.; Duranceau MF.; MacKean G.; Reay T.  **Organizational impact of evidence-informed decision making training initiatives: a case study comparison of two approaches.**  Implementation science : IS 2014;9():53  2014 | | Included | - |
|  | |  |  |
| **#3642 - Courtney 2007**  Courtney KO.; Joe GW.; Rowan-Szal GA.; Simpson DD.  **Using organizational assessment as a tool for program change.**  Journal of substance abuse treatment Sep 2007;33(2):131-7  2007 Sep | | Included | - |
|  | |  |  |
| **#503 - Dagenais 2015**  Dagenais, C.; Some, T. D.; Boileau-Falardeau, M.; McSween-Cadieux, E.; Ridde, V.  **Collaborative development and implementation of a knowledge brokering program to promote research use in Burkina Faso, West Africa**  Glob Health Action 2015;8():26004  2015 | | Included | - |
|  | |  |  |
| **#3881 - Dobbins 2001**  Dobbins, M.; Cockerill, R.; Barnsley, J.  **Factors affecting the utilization of systematic reviews. A study of public health decision makers**  Spring 2001;17(2):203-14  2001 Spring | | Included | - |
|  | |  |  |
| **#1315 - Dobbins 2001**  Dobbins M.; Cockerill R.; Barnsley J.; Ciliska D.  **Factors of the innovation, organization, environment, and individual that predict the influence five systematic reviews had on public health decisions.**  International journal of technology assessment in health care 2001;17(4):467-78  2001 | | Included | - |
|  | |  |  |
| **#2873 - Dobbins 2009**  Dobbins, M.; Hanna, S. E.; Ciliska, D.; Manske, S.; Cameron, R.; Mercer, S. L.; O'Mara, L.; DeCorby, K.; Robeson, P.  **A randomized controlled trial evaluating the impact of knowledge translation and exchange strategies**  Implementation Science 2009;4():61  2009 | | Included | - |
|  | |  |  |
| **#761 - Dopp 2013**  Dopp,C. M.; Graff,M. J.; Rikkert,M. G.; Nijhuis van der Sanden,M. W.; Vernooij-Dassen,M. J.  **Determinants for the effectiveness of implementing an occupational therapy intervention in routine dementia care.**  Implementation Science 2013;8(Journal Article):131  2013 | | Included | - |
|  | |  |  |
| **#1551 - Flanders 2009**  Flanders, S. A.; Kaufman, S. R.; Saint, S.; Parekh, V. I.  **Hospitalists as emerging leaders in patient safety: lessons learned and future directions**  Journal of patient safety 2009;5(1):3-8  2009 | | Included | - |
|  | |  |  |
| **#1349 - Gagliardi 2008**  Gagliardi AR.; Fraser N.; Wright FC.; Lemieux-Charles L.; Davis D.  **Fostering knowledge exchange between researchers and decision-makers: exploring the effectiveness of a mixed-methods approach.**  Health policy (Amsterdam, Netherlands) Apr 2008;86(1):53-63  2008 Apr | | Included | - |
|  | |  |  |
| **#470 - Kitson 2011**  Kitson,Alison; Silverston,Heidi; Wiechula,Rick; Zeitz,Kathryn; Marcoionni,Danni; Page,Tammy  **Clinical nursing leaders', team members' and service managers' experiences of implementing evidence at a local level.**  Journal of Nursing Management 2011;19(4):542-556  2011 | | Included | - |
|  | |  |  |
| **#2005 - Traynor 2014**  Traynor R.; DeCorby K.; Dobbins M.  **Knowledge brokering in public health: a tale of two studies.**  Public health Jun 2014;128(6):533-44  2014 Jun | | Included | - |
|  | |  |  |
| **#1676 - Uneke 2015**  Uneke, Chigozie Jesse; Ndukwe, Chinwendu Daniel; Ezeoha, Abel Abeh; Uro-Chukwu, Henry Chukwuemeka; Ezeonu, Chinonyelum Thecla  **Implementation of a health policy advisory committee as a knowledge translation platform: the Nigeria experience**  International Journal of Health Policy & Management Mar 2015;4(3):161-8  2015 Mar | | Included | - |
|  | |  |  |
| **#3862 - Waqa 2013**  Waqa, Gade; Mavoa, Helen; Snowdon, Wendy; Moodie, Marj; Nadakuitavuki, Rigieta; Mc Cabe, Marita; Swinburn, Boyd  **Participants’ perceptions of a knowledge-brokering strategy to facilitate evidence-informed policy-making in Fiji**  BMC public health 2013;13(1):725  2013 | | Included | - |
|  | |  |  |
| **#3863 - Waqa 2013**  Waqa, Gade; Mavoa, Helen; Snowdon, Wendy; Moodie, Marj; Schultz, Jimaima; McCabe, Marita; Kremer, Peter; Swinburn, Boyd  **Knowledge brokering between researchers and policymakers in Fiji to develop policies to reduce obesity: a process evaluation**  Implement Sci 2013;8():74  2013 | | Included | - |
